# Ligand Engineering of Co‐N_4_ Single‐Atom Catalysts for Highly‐Active and Stable Acidic Oxygen Evolution

**DOI:** 10.1002/advs.202502230

**Published:** 2025-04-30

**Authors:** Taeyoung Jeong, Kiwon Kim, Byung‐Hyun Kim, Sang‐Il Choi, Chang Hyuck Choi, Joonhee Kang, Myeongjin Kim

**Affiliations:** ^1^ School of Energy Engineering Kyungpook National University 80 Daehak‐ro, Bukgu Daegu 41566 Republic of Korea; ^2^ Department of Chemical and Molecular Engineering Hanyang University ERICA 55 Hanyangdaehak‐ro, Sangnok‐gu Ansan‐si Gyeonggi‐do 15588 Republic of Korea; ^3^ Department of Applied Chemistry Center for Bionano Intelligence Education and Research Hanyang University ERICA 55 Hanyangdaehak‐ro, Sangnok‐gu Ansan‐si Gyeonggi‐do 15588 Republic of Korea; ^4^ Department of Chemistry and Green‐Nano Materials Research Center Kyungpook National University 80 Daehak‐ro, Bukgu Daegu 41566 Republic of Korea; ^5^ Department of Chemistry Pohang University of Science and Technology (POSTECH) 77 Cheongam‐ro, Nam‐gu Pohang 37673 Republic of Korea; ^6^ Institute for Convergence Research and Education in Advanced Technology (I‐CREATE) Yonsei University 50 Yonsei‐ro, Seodaemun‐gu Seoul 03722 Republic of Korea; ^7^ Department of Nano Fusion Technology Pusan National University 2 Busandaehak‐ro 63beon‐gil, Geumjeong‐gu Busan 46241 Republic of Korea; ^8^ Department of Nanoenergy Engineering Pusan National University 2 Busandaehak‐ro 63beon‐gil, Geumjeong‐gu Busan 46241 Republic of Korea

**Keywords:** cobalt, orbital rearrangement, oxygen evolution reaction, pyrrolic N, single atom catalyst

## Abstract

The development of stable and efficient single‐atom catalysts (SACs) for the oxygen evolution reaction (OER) in acidic media remains challenging. This work reports a novel NH_3_‐assisted pyrolysis strategy to synthesize Co‐N_4_ SACs with controlled nitrogen coordination environments on crumpled graphene supports. The pyrrolic N_4_‐coordinated Co sites demonstrate superior OER activity compared to their pyridinic counterparts, achieving an overpotential of 351 mV at 10 mA cm^−2^ in 0.5 m H_2_SO_4_. Combined density functional theory calculations and operando X‐ray absorption spectroscopy reveal that the pyrrolic coordination environment facilitates enhanced OH^−^ adsorption and subsequent OER kinetics due to its unique electronic structure and geometric flexibility. A multi‐layered protective mechanism in the pyrrolic system enables exceptional stability during long‐term acidic OER operation, stemming from higher defect formation energy of Co sites and strategic distribution of sacrificial nitrogen species in the graphene network. These findings provide fundamental insights into designing stable single‐atom catalysts for challenging electrochemical applications.

## Graphical abstract

This study highlights the effects of metal – ligand distance on d electron structure and orbital configuration of single atom catalysts (SACs) and their corresponding oxygen evolution reaction (OER) catalytic activity and stability based on the ligand engineering.

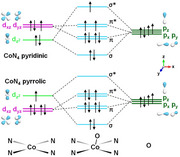

## 1 Introduction

The oxygen evolution reaction (OER) plays a fundamental role in advancing sustainable energy technologies, particularly in applications like water splitting and metal‐air battery systems.^[^ ^1^, ^2^, ^3^, ^4^ ^]^ Despite its critical importance, the OER process faces significant kinetic limitations due to its complex four‐electron transfer mechanism and elevated thermodynamic potential requirement of 1.23 V.^[^ ^5^, ^6^ ^]^ Traditional noble metal catalysts, specifically RuO_2_ and IrO_2_, have demonstrated superior OER activity but their scarcity and high cost have driven researchers to explore alternative solutions using earth‐abundant elements.^[^ ^7^, ^8^, ^9^ ^]^ While significant progress has been made in developing transition metal (TM)‐based OER catalysts, their performance metrics and atomic utilization efficiency remain suboptimal. Single‐atom catalysts (SACs) have emerged as a groundbreaking approach in electrocatalysis, offering maximized atomic efficiency through isolated metal active sites.^[^ ^10^, ^11^, ^12^, ^13^ ^]^ Of particular interest is the development of SACs incorporating porphyrin‐like architectures, which draw inspiration from natural systems such as the oxygen‐evolving complex in photosynthesis and oxygen‐carrying proteins in biological systems.^[^ ^14^, ^15^ ^]^ The distinctive structure of porphyrins, characterized by four coordinating pyrrolic nitrogen (N) atoms arranged in a conjugated heterocyclic framework, provides unique advantages for OER catalysis. These pyrrolic nitrogen‐based coordination environments demonstrate remarkable capability in stabilizing transition metals in high oxidation states while facilitating the critical O─O bond formation step.^[^ ^16^, ^17^, ^18^, ^19^, ^20^ ^]^ Consequently, the strategic incorporation of pyrrole units as coordinating ligands for transition metal centers represents a promising design approach for next‐generation SACs.

To develop the highly active single atomic sites by using TM‐pyrrolic N_4_ moiety, extensive research efforts have focused on developing small‐molecule metalloporphyrin electrocatalysts, owing to their precisely defined transition metal‐pyrrolic N_4_ coordination environments, which allow homogeneous control over the primary coordination sphere and enable effective catalytic active sites.^[^ ^21^, ^22^, ^23^ ^]^ These molecular catalysts exhibit remarkable activity and selectivity across diverse electrochemical processes, attributed to their highly ordered metal‐pyrrolic N active sites.^[^ ^24^, ^25^, ^26^ ^]^ However, the practical implementation of these organometallic systems faces significant challenges, particularly regarding their stability and recyclability, as the metal centers tend to detach during operation.^[^ ^27^, ^28^ ^]^ To address these limitations, researchers have explored the integration of single‐atom transition metal sites within carbon‐nitrogen frameworks (M‐N‐C catalysts).^[^ ^29^ ^]^ These M‐N‐C catalysts demonstrate enhanced operational stability, making them more suitable for practical applications. While M‐N‐C systems offer the flexibility to modulate catalytic performance through variations in N doping levels and carbon defect structures, their synthesis typically requires high‐temperature pyrolysis processing.^[^ ^30^, ^31^, ^32^ ^]^ This thermal requirement presents a significant challenge as pyrrolic nitrogen groups, crucial for mimicking porphyrin‐like coordination environments, undergo thermal transformation to graphitic or pyridinic nitrogen configurations during high‐temperature treatment.^[^ ^33^, ^34^ ^]^ Despite extensive efforts to recreate porphyrin‐inspired structures within M‐N‐C materials, current approaches have achieved only partial success in replicating the precise coordination environment of molecular porphyrins. Most existing methods result in approximations of the desired metal‐nitrogen bonding arrangement rather than exact structural analogs.^[^ ^35^, ^36^, ^37^, ^38^, ^39^, ^40^, ^41^ ^]^ Therefore, the formation of homogeneously isolated TM‐pyrrolic N_4_ moiety in M‐N‐C still remains a great challenge, and there have been very few attempts to construct TM‐pyrrolic N_4_ moiety on carbon matrix by ammonia (NH_3_) assisted pyrolysis.^[^ ^42^, ^43^ ^]^

Herein, we demonstrate a novel synthesis approach using ammonia‐assisted pyrolysis to successfully incorporate cobalt single atomic sites with pyrrolic N_4_ coordination environments into carbon networks. We provide comparative insights into the OER performance between Co‐pyrrolic N_4_ and Co‐pyridinic N_4_ structures, revealing crucial structure‐activity relationships. Density Functional Theory (DFT) reveals that the extended metal‐ligand bond distance characteristic of pyrrolic N coordination generates an unstable $d_{z^{2}}$ orbital configuration, resulting in elevated energy levels within the square planar geometry. This electronic structure leads to vacant σ* molecular orbitals during oxygen adsorption, enhancing the binding capabilities of O* intermediates. Our experimental findings validate the computational predictions, demonstrating superior OER performance of Co‐pyrrolic N_4_ supported on crumpled graphene (Pyrrolic CoN_4_‐CG) compared to its pyridinic counterpart (Pyridinic CoN_4_‐CG) in acidic conditions (0.5 M H_2_SO_4_). The pyrrolic system achieves a current density of 10 mA cm^−2^ at an overpotential of 351 mV, highlighting its enhanced catalytic efficiency. To elucidate the mechanistic origins of this superior activity, *operando* X‐ray absorption spectroscopy (*Operando* XAS) measurements reveal that isolated Co centers in Pyrrolic CoN_4_‐CG undergo more favorable oxidation processes compared to Pyridinic CoN_4_‐CG. The higher oxidation state means the efficient OH^−^ adsorption on Co single atomic sites, which promotes faster OER kinetics by accelerating the progression through subsequent reaction steps. This work presents the first comprehensive investigation linking electronic structure to OER catalytic performance in ligand‐engineered SACs. Our findings provide fundamental design principles for developing highly efficient SAC systems and introduce a novel strategy for optimizing their catalytic performance through ligand environment control.

## 2 Results and Discussion

**Figure 1:**
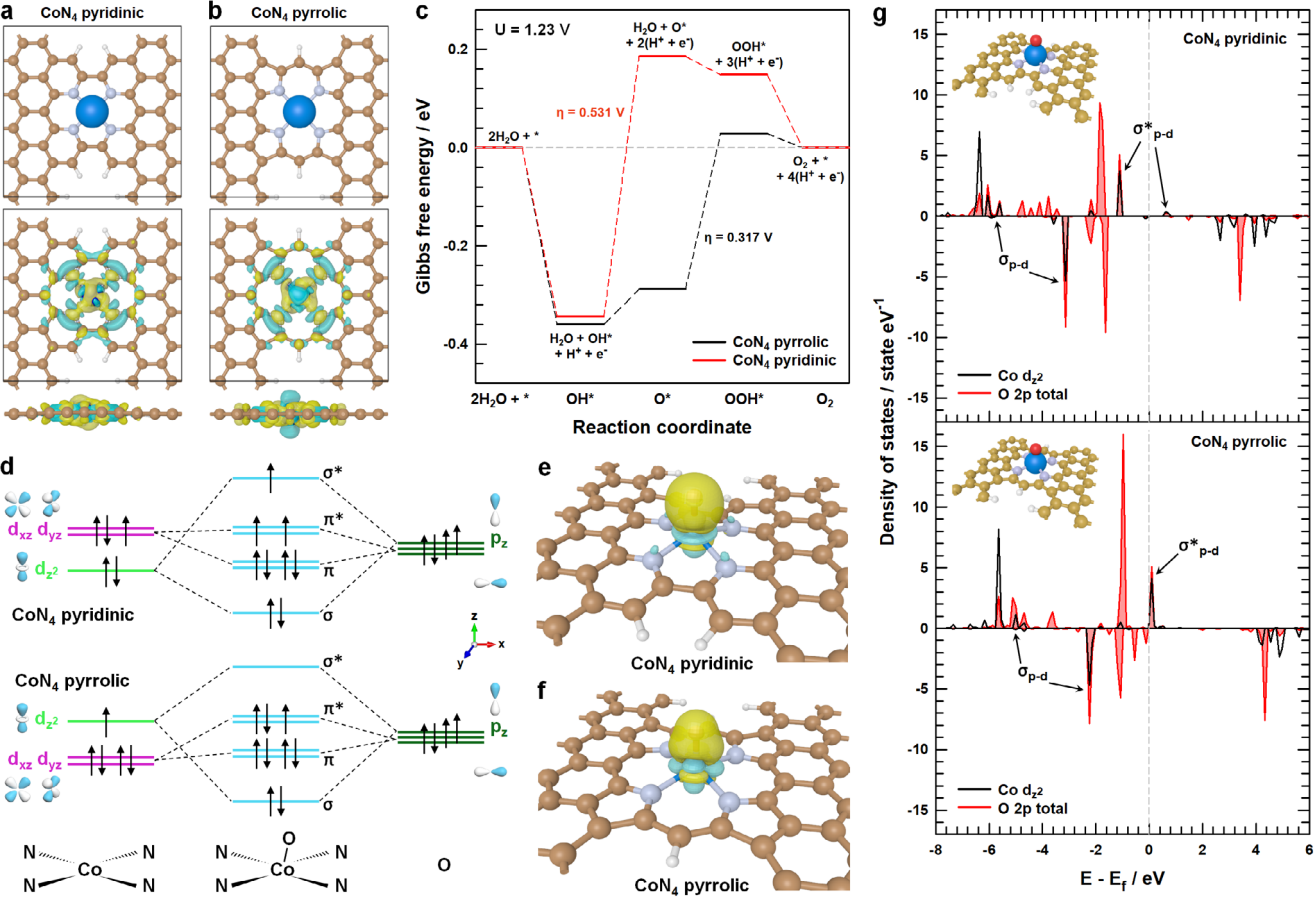
The optimized structure and charge density difference of a) CoN_4_ pyridinic and b) CoN_4_ pyrrolic. (blue, Co; light blue, N; brown, C; white, H). Yellow and cyan isosurfaces (0.015 e Å^−3^) denote the electron accumulation and depletion, respectively. c) Free energy diagram of the oxygen evolution reaction for CoN_4_ pyrrolic and CoN_4_ pyridinic. (U = 1.23 V) d) The orbital interactions between Co^2+^ cations in CoN_4_ pyrrolic and CoN_4_ pyridinic models and O. e,f) Charge density difference of oxygen‐adsorbed CoN_4_ pyridinic and CoN_4_ pyrrolic. g) The PDOS of Co d_z2_ and O 2p for CoN_4_ pyridinic and CoN_4_ pyrrolic after the O adsorption.

Comprehensive DFT calculations were conducted to understand the mechanistic advantages of pyrrolic N ligand systems in SACs for OER, investigating atomic‐scale reaction mechanisms and rate‐determining step (RDS). Computational models were developed comparing Co single atomic sites coordinated within pyridinic (CoN_4_ pyridinic) and pyrrolic (CoN_4_ pyrrolic) nitrogen‐doped carbon frameworks, which exhibit distinct metal‐ligand bond distances (**Figure** **1**a,b).^[^ ^42^, ^44^ ^]^ Charge density distributions revealed that while both nitrogen environments form strong sigma bonds in their respective CoN_4_ structures, the pyrrolic configuration uniquely displays charge depletion along the z‐axis of the cobalt center. Figure 1c shows the free energy diagram of the four‐electron pathway OER at equilibrium potential (1.23 V) by the standard hydrogen electrode method.^[^ ^45^ ^]^ The kinetic analysis identifies different RDS between the two configurations, with the pyrrolic CoN_4_ structure exhibits an overpotential of 0.317 V for OOH* adsorption, while the pyridinic CoN_4_ configuration shows a higher overpotential of 0.531 V for O* adsorption, demonstrating the enhanced OER activity of the pyrrolic configuration. Electronic structure and d‐orbital energy distributions of Co^2+^ in both coordination environments were analyzed using projected density of states (PDOS) to rationalize this performance difference (Figure S1, Supporting Information). The shorter Co─N bond length in the pyridinic configuration induces an orbital inversion between $d_{z^{2}}$ and e_g_ (*d_xz_* and *d_yz_*) levels when transitioning from pyrrolic to pyridinic coordination, significantly affecting electron occupancy in the $d_{z^{2}}$ orbital. This electronic structure difference profoundly impacts oxygen intermediate binding. In the pyridinic system, high electron density in the Co $d_{z^{2}}$ orbital results in occupied sigma antibonding (σ*_p–d_) molecular orbital upon O adsorption, weakening the oxygen binding and making O* adsorption rate‐limiting. Conversely, the pyrrolic system's lower $d_{z^{2}}$ electron occupancy enables formation of empty σ*_p–d_ states during oxygen adsorption, promoting stronger O binding (Figure 1d). Charge density difference analysis provides additional support for these findings through the observation of distinct oxygen binding modes, with the pyridinic system showing σ‐bonding and the pyrrolic configuration displaying π‐bonding behavior (Figure 1e,f; Figure S2, Supporting Information). The PDOS analysis of Co‐O hybrid orbitals reveals that the pyrrolic system's higher‐energy σ*_p–d_ states enable stronger oxygen adsorption, which accounts for the enhanced OER kinetics observed in pyrrolic N‐coordinated structures (Figure 1g).

**Figure 2:**
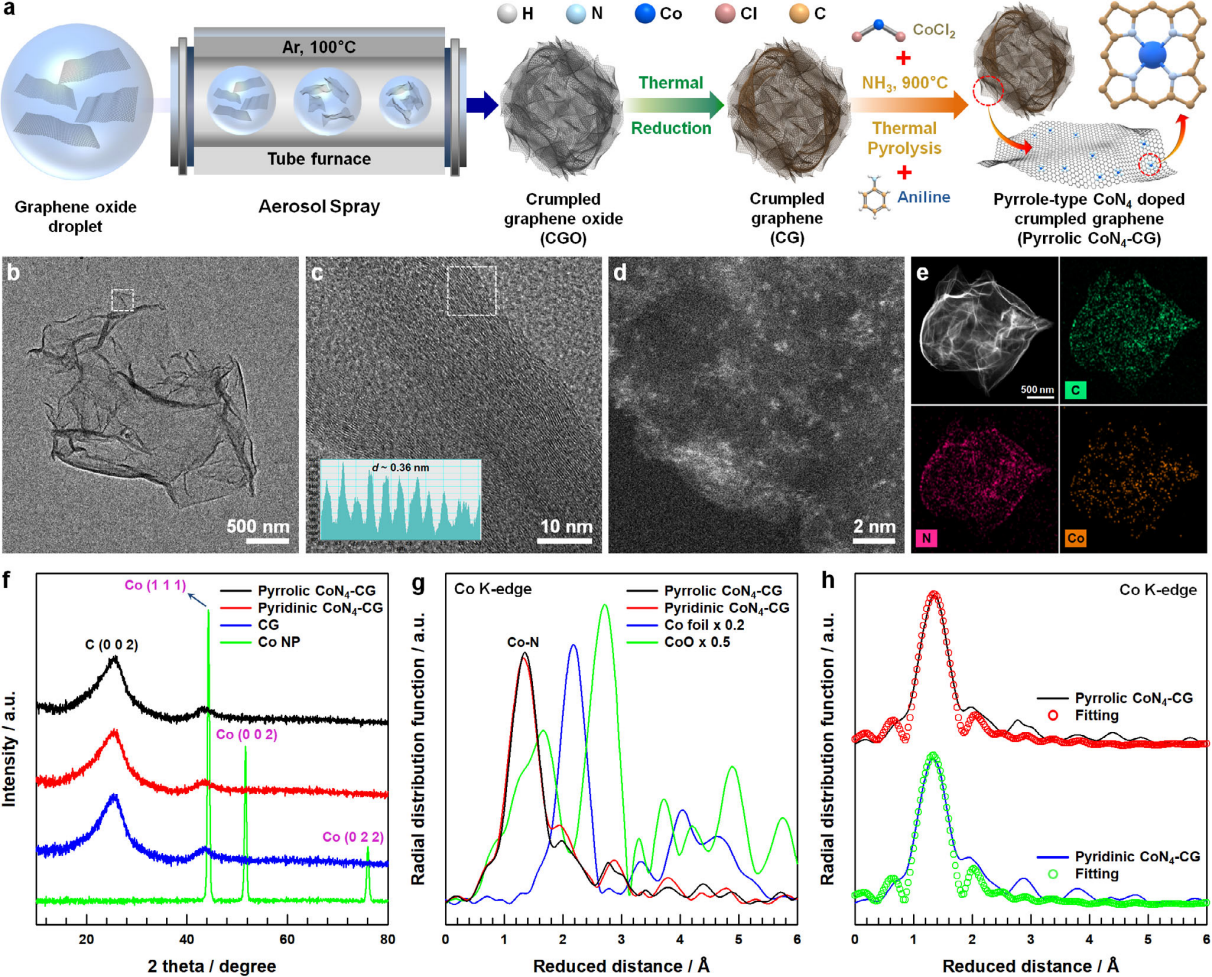
a) Synthesis process of Pyrrolic CoN_4_‐CG. b,c) High‐resolution transmission electron microscopy (HR‐TEM) images of Pyrrolic CoN_4_‐CG at different magnifications. d) High‐angle annular dark field scanning transmission electron microscopy (HAADF‐STEM) image of Pyrrolic CoN_4_‐CG. e) Energy dispersive X‐ray analysis (EDX) mapping of Pyrrolic CoN_4_‐CG. f) X‐ray powder diffraction (XRD) of CG, Co NP, Pyrrolic CoN_4_‐CG and Pyridinic CoN_4_‐CG. g,h) Co K‐edge extended X‐ray absorption fine structure (EXAFS) spectra and EXAFS Co K‐edge fitting of Pyrrolic CoN_4_‐CG and Pyridinic CoN_4_‐CG.

Experimental validation of the pyrrolic N ligand system's advantages for OER was conducted through the synthesis of distinct CoN_4_ sites on crumpled graphene (CG) supports. The preparation of pyrrolic (Pyrrolic CoN_4_‐CG) and pyridinic (Pyridinic CoN_4_‐CG) CoN_4_ sites utilized polyaniline and Co single atom precursors, with the key difference being the presence or absence of NH_3_ during pyrolysis, which controls carbon atom removal from the graphene network (**Figure** **2**a). Morphological characterization of Pyrrolic CoN_4_‐CG through field emission scanning electron microscopy (FE‐SEM) revealed a successfully formed 3D crumpled structure with characteristic open pores (Figure S3, Supporting Information). Field emission transmission electron microscopy (FE‐TEM) analysis confirmed the presence of ridge structures resulting from compression during aerosol spraying (Figure 2b; Figure S4, Supporting Information). High‐magnification TEM image showed an expanded graphene layer with a d‐spacing of ≈0.36 nm, notably larger than typical graphite (0.336 nm) (Figure 2c).^[^ ^46^ ^]^ High‐angle annular dark field scanning transmission electron microscopy (HAADF‐STEM) image identified bright spots corresponding to individual Co single atoms, and energy dispersive X‐ray (EDX) mapping demonstrated uniform distribution of Co, N, and C throughout the structure (Figure 2d,e). Similar morphological characteristics were also observed for Pyridinic CoN_4_‐CG (Figure S5, Supporting Information). X‐ray powder diffraction (XRD) patterns exhibited broad and low‐intensity (002) peaks characteristic of graphene structures for both materials.^[^ ^47^ ^]^ The absence of metallic Co diffraction peaks confirmed the atomic dispersion of Co species within the graphene framework (Figure 2f). The Co K‐edge extended X‐ray absorption fine structure (EXAFS) analysis revealed a prominent peak at ≈1.34 Å attributed to Co‐N coordination, with no evidence of Co‐Co interactions typical of Co foil or metallic clusters (Figure 2g).^[^ ^48^ ^]^ EXAFS fitting analysis revealed that Co single atom in Pyrrolic CoN_4_‐CG and Pyridinic CoN_4_‐CG exhibit 4.08 and 4.05 of coordination number with surrounding N, representing Co atom is bonded with four N atoms (Figure 2h). A significant structural difference lies in the Co─N bond distances, where Pyrrolic CoN_4_‐CG exhibits longer interatomic spacing (2.07 Å) compared to Pyridinic CoN_4_‐CG (1.89 Å). This variation in bond length stems from the underlying geometric differences between the pentagonal carbon rings containing pyrrolic nitrogen and the hexagonal carbon rings containing pyridinic nitrogen.

**Figure 3:**
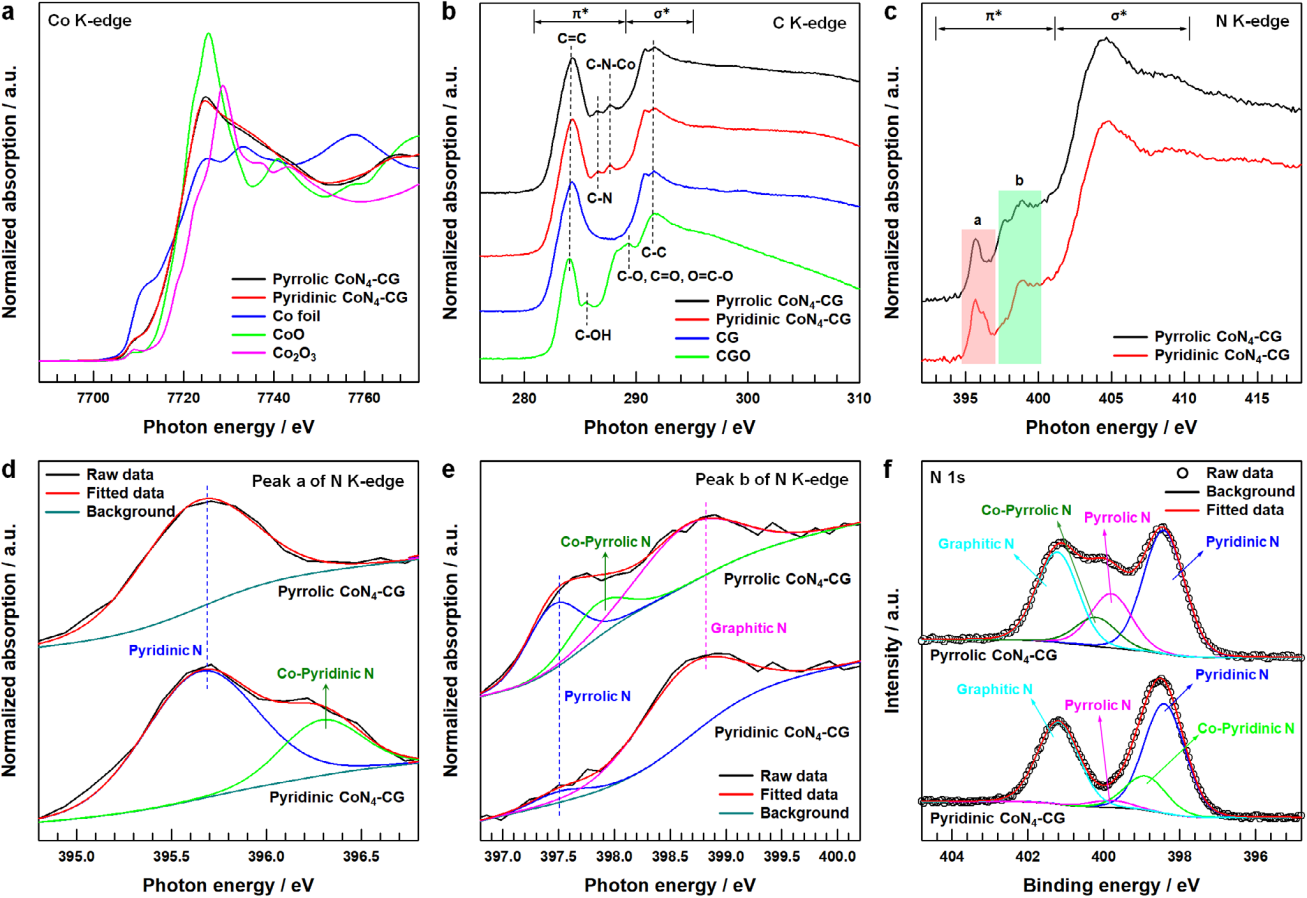
a) Co K‐edge X‐ray absorption near edge structure (XANES) spectra of Co foil, CoO, Co_2_O_3_, Pyrrolic CoN_4_‐CG and Pyridinic CoN_4_‐CG. b,c) C K‐edge soft X‐ray absorption near‐edge spectroscopy (sXAS) spectra and N K‐edge sXAS spectra of CGO, CG, Pyrrolic CoN_4_‐CG, and Pyridinic CoN_4_‐CG. d,e) Peak a and Peak b of N K‐edge sXAS spectra of Pyrrolic CoN_4_‐CG and Pyridinic CoN_4_‐CG. f) N 1s X‐ray photoelectron spectroscopy (XPS) spectra of Pyrrolic CoN_4_‐CG and Pyridinic CoN_4_‐CG.

The electronic state and local chemical environment of Co atoms in both Pyrrolic CoN_4_‐CG and Pyridinic CoN_4_‐CG were investigated using Co K‐edge X‐ray absorption near edge structure (XANES) analysis (**Figure** **3**a). Both materials display characteristic edge peaks ≈7725 eV, typical of CoN_4_ coordination environments.^[^ ^49^ ^]^ Analysis of the first derivative Co K‐edge XANES spectra revealed Co valence states of 2.048 and 2.009 for Pyrrolic CoN_4_‐CG and Pyridinic CoN_4_‐CG, respectively (Figure S6, Supporting Information).^[^ ^50^ ^]^ In addition, the Co 2p spectra of Pyrrolic CoN_4_‐CG and Pyridinic CoN_4_‐CG (Figure S7, Supporting Information) show peaks at 796.26 eV and 780.93 eV, corresponding to the Co 2p_1/2_ and 2p_3/2_ orbitals of a Co^2+^ species, respectively, further confirming the Co^2+^ oxidation states in CoN_4_ moiety. Soft X‐ray absorption near‐edge spectroscopy (sXAS) provided detailed insights into the local chemical configurations. The C K‐edge sXAS spectra (Figure 3b) exhibited graphene‐characteristic features including π*_C = C_ (∼284 eV) and σ*_C‐C_ (∼291.8 eV) peaks across all samples.^[^ ^51^ ^]^ While CGO displayed various oxygen‐containing functional groups, CG showed predominantly C═C and C─C peaks with enhanced C═C intensity, indicating successful thermal reduction. Both CoN_4_ materials demonstrated additional peaks at 286.4 and 287.9 eV, attributed to C─N and C─N─Co bonds respectively, confirming successful incorporation of Co and N into the graphene framework. N K‐edge spectra analysis of Pyrrolic CoN_4_‐CG (Figure 3c) demonstrated two distinct regions with π* ranging from 393.0 to 401.0 eV and σ* spanning 401.0 to 410.0 eV.^[^ ^42^, ^52^, ^53^ ^]^ The π* region exhibited two prominent resonances, labeled as peaks ‘a’ and ‘b’, which correspond to pyridinic and pyrrolic/graphitic nitrogen transitions. Detailed peak fitting of the ‘a’ region (Figure 3d) indicated that Pyridinic CoN_4_‐CG contains both pyridinic N (395.7 eV) and Co‐pyridinic N (396.4 eV) components, while Pyrrolic CoN_4_‐CG displays only pyridinic N. Examination of peak ‘b’ (Figure 3e) showed that Pyrrolic CoN_4_‐CG comprises pyrrolic N (397.5 eV), Co‐pyrrolic N (398.0 eV), and graphitic N (398.8 eV) components, whereas Pyridinic CoN_4_‐CG exhibits only pyrrolic and graphitic N signals. X‐ray photoelectron spectroscopy (XPS) N 1s spectra (Figure 3f) further validated these findings. Pyrrolic CoN_4_‐CG exhibited four distinct peaks corresponding to pyridinic N (398.42 eV), pyrrolic N (399.8 eV), Co‐pyrrolic N (400.2 eV), and graphitic N (401.2 eV). In contrast, Pyridinic CoN_4_‐CG showed a characteristic Co‐pyridinic N peak at 398.9 eV instead of the Co‐pyrrolic N signal, corroborating the sXAS results.^[^ ^53^, ^54^ ^]^

**Figure 4:**
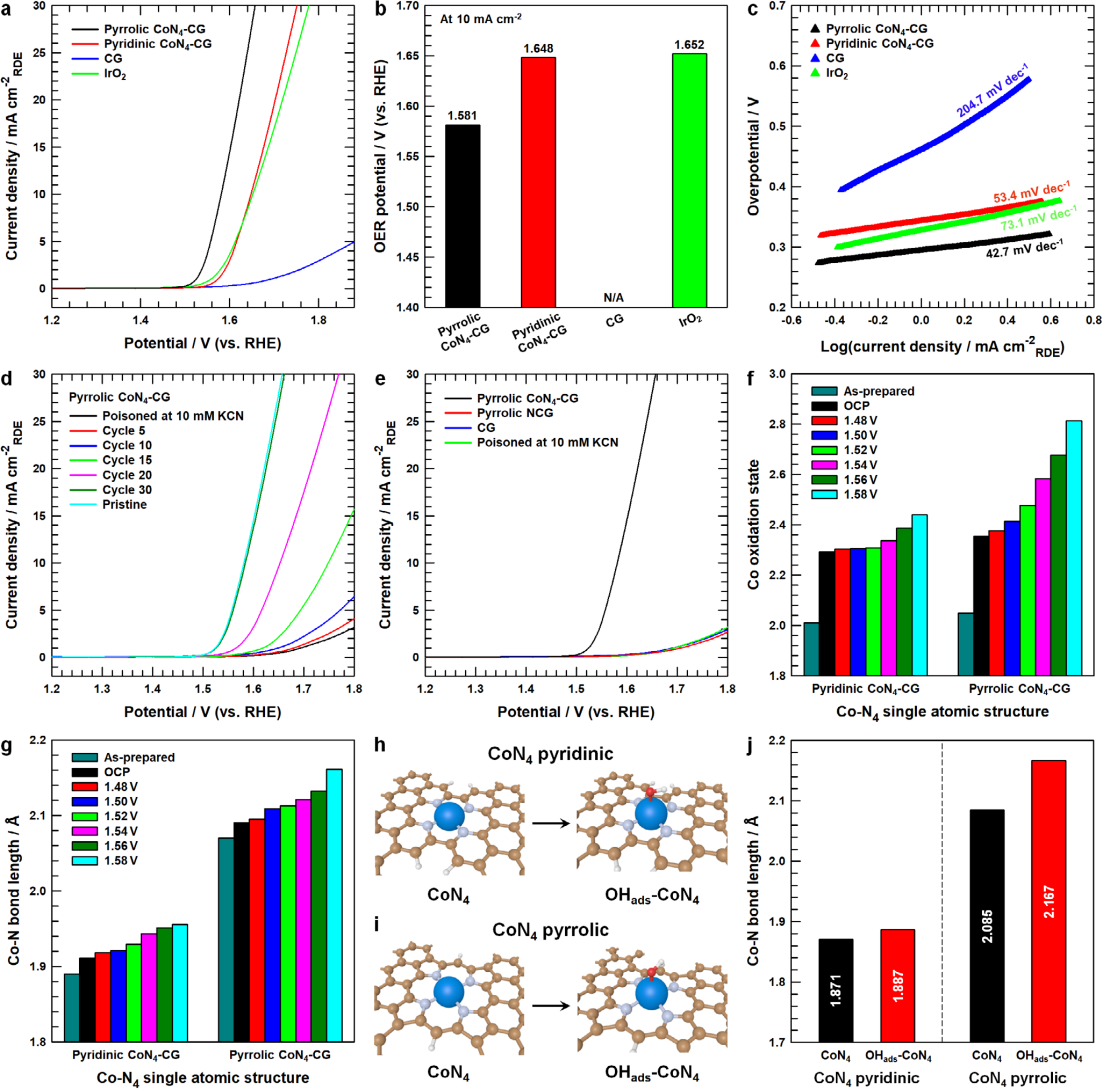
a) *iR*‐corrected OER polarization curves for CG, Pyrrolic CoN_4_‐CG, Pyridinic CoN_4_‐CG and IrO_2_ at a scan rate of 5 mV s^−1^ and a rotation rate of 1600 rpm in O_2_‐saturated 0.5 m H_2_SO_4_ electrolyte. b) Comparison of the OER potentials required to achieve a current density of 10 mA cm^−2^, and c) corresponding Tafel plots for CG, Pyrrolic CoN_4_‐CG, Pyridinic CoN_4_‐CG and IrO_2_. d) LSV polarization curves of KCN‐poisoning and recovery experiments of Pyrrolic CoN_4_‐CG in O_2_‐saturated 0.5 m H_2_SO_4_ electrolyte. e) Comparison of LSV polarization curves for Pyrrolic CoN_4_‐CG, Pyrrolic NCG, CG, and KCN‐poisoned (with 10 mm KCN) Pyrrolic CoN_4_‐CG. f) Changes of Co oxidation states during OER analyzed by Operando XANES Co K‐edge spectra. g) Changes of Co─N bond length during OER analyzed by Operando EXAFS Co K‐edge spectra. Relaxed structures after OH adsorption for h) CoN_4_ pyridinic and i) CoN_4_ pyrrolic, respectively. j) Calculated Co─N bond length for CoN_4_ pyridinic and pyrrolic models with and without OH adsorption.

The OER catalytic performance of both Pyrrolic CoN_4_‐CG and Pyridinic CoN_4_‐CG was evaluated under acidic conditions (O_2_‐saturated 0.5 m H_2_SO_4_) using rotating disk electrode (RDE) measurements at a rotation rate of 1600 rpm and a scan rate of 5 mV s^−1^. The Pyrrolic CoN_4_‐CG demonstrated superior performance with the lowest overpotential and Tafel slope than those of Pyridinic CoN_4_‐CG and benchmark IrO_2_, indicating that the incorporation of Co‐pyrrolic N_4_ moieties significantly enhances OER activity (**Figure** **4**a–c). The role of CoN_4_ active sites was investigated through CN‐ poisoning experiments using 10 mm KCN in O2‐saturated 0.5 m H_2_SO_4_ (Figure 4d; Figures S8 and S9, Supporting Information). Under acidic conditions, CN^−^ selectively deactivates metal catalytic centers.^[^ ^55^, ^56^ ^]^ Both Pyrrolic CoN_4_‐CG and Pyridinic CoN_4_‐CG showed immediate activity loss upon KCN addition, followed by gradual recovery over 30 cycles until reaching initial performance levels, confirming isolated Co atoms as the primary catalytic active centers. To differentiate between the effects of Co sites and nitrogen functionalization, control catalysts were synthesized without Co precursors, producing pyrrole‐type (Pyrrolic NCG) and pyridine‐type (Pyridinic NCG) nitrogen‐doped crumpled graphene (Figures S10 and S11, Supporting Information). These N‐doped CG catalysts displayed OER activities comparable to unmodified CG, despite their different nitrogen content and speciation, indicating negligible contribution from nitrogen functionalization alone to OER activity (Figure 4e; Figures S12 and S13, Supporting Information). Furthermore, the KCN‐poisoned Pyrrolic CoN_4_‐CG and Pyridinic CoN_4_‐CG showed activity levels similar to CG, Pyrrolic NCG and Pyridinic NCG, definitively establishing isolated Co atoms as the primary active sites in both pyrrolic and pyridinic configurations. Moreover, Pyrrolic CoN_4_‐CG achieved superior mass‐specific and surface area‐normalized OER activities despite having lower specific surface area and Co loading compared to Pyridinic CoN_4_‐CG, emphasizing the crucial role of pyrrole‐type nitrogen coordination in enhancing catalytic performance (Figure S14, Supporting Information).

The superior intrinsic OER activity of pyrrole‐type Co‐N_4_ sites was investigated through operando XAS analysis under actual reaction conditions in O_2_‐saturated 0.5 m H_2_SO_4_. The measurements were conducted across various applied potentials, starting from open‐circuit potential (OCP). The Co K‐edge XANES spectra at OCP indicated slight oxidation of Co single atoms in both Pyrrolic CoN_4_‐CG and Pyridinic CoN_4_‐CG due to dissolved oxygen in the electrolyte (Figures S15 and S16, Supporting Information). Notably, as the applied potential increased from 1.48 to 1.58 V (vs RHE), Pyrrolic CoN_4_‐CG exhibited a more pronounced positive shift in Co K‐edge XANES spectra compared to Pyridinic CoN_4_‐CG. This observation indicates more extensive oxidation of Co sites to higher valence states in the pyrrolic configuration, leading to enhanced OH^−^ adsorption capabilities (Figure 4f). Local structural environment around Co centers was monitored through operando EXAFS analysis (Figure S17, Supporting Information). While both samples maintained their primary Co─N coordination shell, increasing applied potential resulted in gradual increase of Co─N bond length, indicating chemical adsorption of OH^−^ intermediates (Figure 4g). The elongation of Co─N bonds during OH adsorption results from coupled electronic and geometric factors. The electronic redistribution induced by OH coordination to the Co center, particularly involving the $d_{z^{2}}$ orbital, leads to decreased electron density in the Co─N bonds.^[^ ^57^ ^]^ This electronic effect is accompanied by structural distortion where the Co center shifts slightly out of plane along the z‐axis to accommodate the OH adsorbate.^[^ ^58^ ^]^ Importantly, Pyrrolic CoN_4_‐CG exhibits a notably more pronounced increase in Co─N bond length than Pyridinic CoN_4_‐CG during OER process. The pyrrolic coordination environment, with its pentagonal geometry, inherently provides greater structural flexibility compared to the more rigid hexagonal arrangement in the pyridinic system. This enhanced flexibility allows for more pronounced geometric distortion upon OH adsorption.^[^ ^59^ ^]^ Additionally, the pentagonal structure's inherent strain enables more efficient electronic redistribution when the Co center interacts with OH, leading to a more substantial bond elongation. Finally, the pyrrolic N configuration facilitates substantial increases in Co oxidation state (Figure 4f). Although this higher oxidation state results in a contracted Co ionic radius, it simultaneously amplifies the z‐directional displacement through intensified electrostatic attraction with OH^−^ species.^[^ ^58^ ^]^ Computational modeling of OH^−^ adsorption structures corroborated these experimental findings (Figure 4h–j). The calculations revealed a more significant increase in Co─N bond length upon OH^−^ adsorption in the pyrrolic configuration compared to the pyridinic model. This result aligns with the operando EXAFS observations and can be attributed to the electron‐deficient nature of the $d_{z^{2}}$ orbital in pyrrolic Co sites, which promotes favorable electrostatic interactions with OH^−^ species.

**Figure 5:**
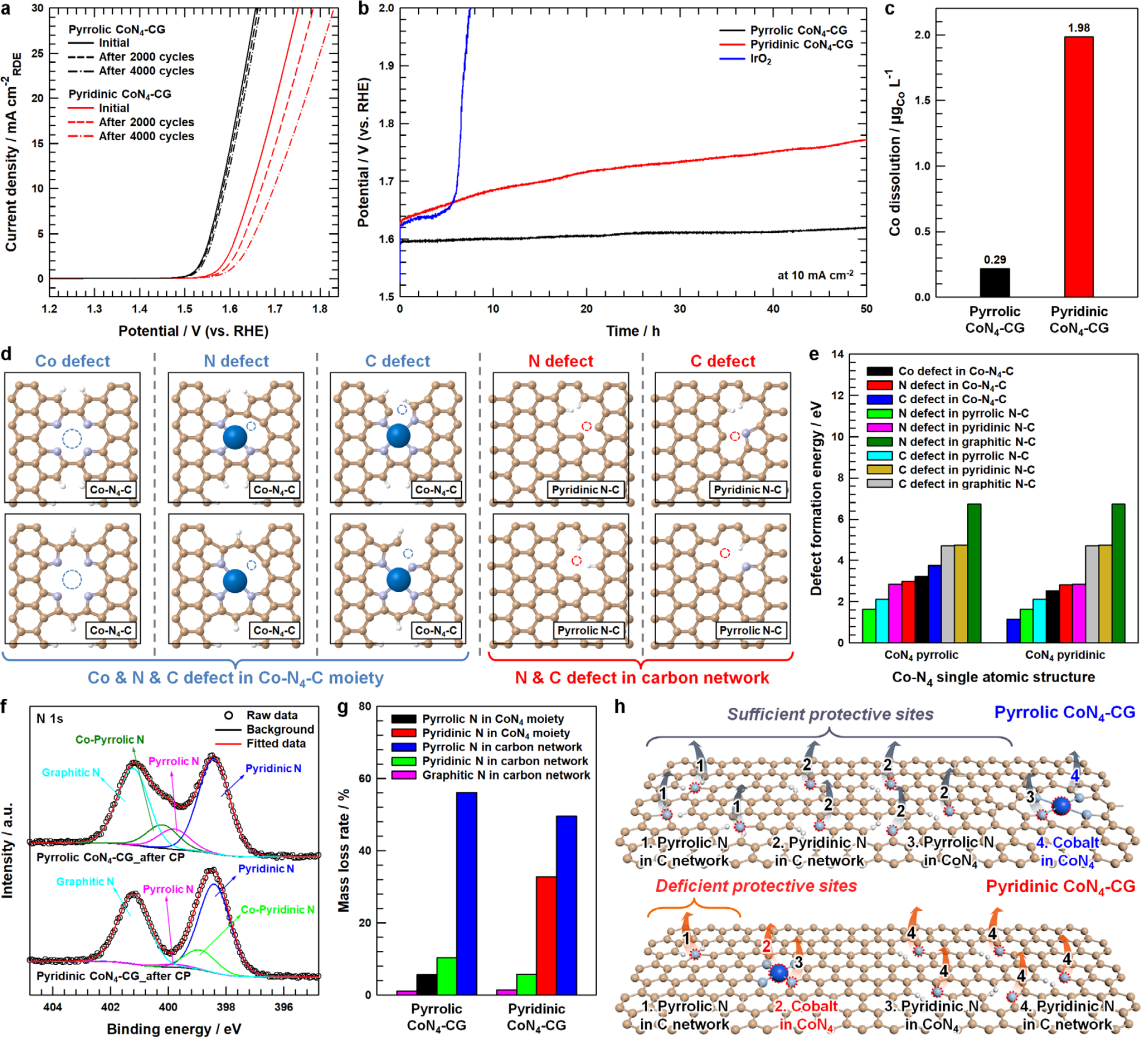
a) OER polarization curves of Pyrrolic CoN_4_‐CG and Pyridinic CoN_4_‐CG before and after 2000 and 4000 CV cycles. b) Comparison of chromopotentiometric (CP) responses of Pyrrolic CoN_4_‐CG, Pyridinic CoN_4_‐CG and IrO_2_ at a constant current density of 10 mA cm^−2^ over 50 h. c) Comparison of the Co leaching amount from Pyrrolic CoN_4_‐CG and Pyridinic CoN_4_‐CG into testing solution during 50 h of stability test. d) Atomic defect model systems in the Co‐N_4_‐C moiety and N‐doped carbon network for evaluating structural stability using DFT calculations. e) Calculated defect formation energies of Co, N, and C in CoN_4_ pyrrolic and CoN_4_ pyridinic. f) XPS N 1s spectra of post‐chronopotentiometric samples, labeled as Pyrrolic CoN_4_‐CG_after CP and Pyridinic CoN_4_‐CG_after CP. g) Mass loss rate of specific N species in Pyrrolic CoN_4_‐CG and Pyridinic CoN_4_‐CG after 50 h of stability test. h) The Co and specific N species dissolution mechanism in Pyrrolic CoN_4_‐CG and Pyridinic CoN_4_‐CG during acidic OER.

Long‐term stability assessments revealed remarkable durability of the pyrrole‐type Co‐N_4_ structure under acidic conditions (**Figure** **5**a,b). Durability evaluations through cyclic voltammetry demonstrated that Pyrrolic CoN_4_‐CG maintained its catalytic performance with minimal degradation over 4000 cycles, while Pyridinic CoN_4_‐CG showed significant activity loss. Chronopotentiometry (CP) measurements at a constant current density of 10 mA cm^−2^ over 50 h further confirmed this trend, with Pyrrolic CoN_4_‐CG exhibiting superior stability compared to the notable performance decay observed in Pyridinic CoN_4_‐CG, consistent with post‐CV linear sweep voltammetry results. Given that degradation of catalytic activity mainly stems from active site dissolution, the influence of nitrogen coordination environment on Co dissolution during OER operation was investigated. Quantitative analysis of Co leaching during the 50 h of CP test was performed using inductively coupled plasma mass spectrometry (ICP‐MS) (Figure 5c). The measurements revealed substantially higher Co dissolution in Pyridinic CoN_4_‐CG (1.98 µg_Co_ L^−1^) compared to Pyrrolic CoN_4_‐CG (0.29 µg_Co_ L^−1^), with ≈6.83‐fold difference in leaching rates between the two catalysts. DFT calculations were employed to investigate the mechanistic basis for the dramatic difference in Co leaching between pyrrolic and pyridinic nitrogen coordination environments, focusing on defect formation energies of Co, C, and N atoms. In both Pyridinic and Pyrrolic CoN_4_‐CG catalysts, comprehensive defect formation energy calculations were performed to account for the diverse chemical environments of nitrogen and carbon atoms. These environments include: N atoms directly coordinated to Co in the Co‐N_4_ moiety (either pyrrolic or pyridinic N), C atoms adjacent to these coordinating N, N atoms doped into the graphene network but not participating in Co coordination (including pyrrolic, pyridinic, and graphitic N), and their neighboring C atoms (Figure 5d; Figure S18, Supporting Information). The defect formation energy of the Co active site in the CoN_4_ pyrrolic is 3.24 eV, which is significantly higher compared to Co in the CoN_4_ pyridinic (2.53 eV), indicating that the Co active site remains more stable in the CoN_4_ pyrrolic (Figure 5e). While the 0.71 eV difference in defect formation energies alone cannot fully explain the more than six‐fold difference in Co dissolution rates, a comprehensive analysis of defect formation energetics across various N and C environments provides deeper mechanistic insights. In the CoN_4_ pyrrolic system, four distinct components exhibit lower defect formation energies than Co, including pyrrolic N in the graphene network, its adjacent C atoms, pyridinic N in the graphene network, and pyrrolic N ligands within the Co‐pyrrolic N_4_ moiety. In contrast, the CoN_4_ pyridinic system shows only three components with lower defect formation energies, specifically C atoms adjacent to the Co‐pyridinic N_4_ moiety, pyrrolic N in the graphene network, and its neighboring C atoms.

This phenomenon reveals a critical protective mechanism where the CoN_4_ pyrrolic system possesses more sacrificial components that must undergo dissolution before Co leaching can occur. These components effectively serve as structural barriers, providing additional protection against Co dissolution. The greater number of such protective components in the CoN_4_ pyrrolic system significantly delays Co leaching compared to the CoN_4_ pyridinic configuration. Furthermore, the enhanced stability is further amplified by the quantitative differences in protective components between the two catalysts. Pyrrolic CoN_4_‐CG contains substantially higher concentrations of pyrrolic N in the graphene network and its adjacent C atoms, which serve as crucial structural barriers (Figures 3f and 5f; Tables S1–S3, Supporting Information). Additionally, pyridinic N in the graphene network, which represents the dominant nitrogen species, functions as an additional protective component in Pyrrolic CoN_4_‐CG but not in Pyridinic CoN_4_‐CG. This combination of higher absolute quantities of protective components and the involvement of abundant pyridinic N as a structural barrier significantly accelerates the protective effect against Co dissolution in the Pyrrolic CoN_4_‐CG. Therefore, Pyrrolic CoN_4_‐CG exhibits higher mass loss rates for pyridinic N and pyrrolic N in the graphene network, which serve as primary structural barriers, compared to Pyridinic CoN_4_‐CG after 50 h of CP operation (Figure 5g). However, the pyrrolic N ligands within the Co‐N_4_ moiety show lower mass loss rates in Pyrrolic CoN_4_‐CG. This selective degradation pattern, where sacrificial nitrogen species in the graphene network preferentially dissolve while preserving the integrity of the Co‐pyrrolic N_4_ moiety, enables sustained catalytic performance during long‐term operation. Comprehensively, Pyrrolic CoN_4_‐CG exhibits exceptional stability against active site dissolution under acidic OER conditions through a multi‐layered protective mechanism. This enhanced durability stems from both the higher defect formation energy of Co sites and the presence of multiple sacrificial components in the graphene network. The system's abundant pyrrolic and pyridinic N species serve as structural barriers, undergoing preferential dissolution before Co leaching can occur (Figure 5h). Conversely, Pyridinic CoN_4_‐CG demonstrates accelerated degradation due to its limited number of protective components, where the sparse distribution of pyrrolic N in the graphene network provides insufficient protection, leading to rapid Co dissolution and subsequent structural collapse of the active sites.

## 3 Conclusion

In summary, we have successfully demonstrated a strategy for synthesizing highly stable and active Co‐N_4_ single‐atom catalysts through NH_3_‐assisted pyrolysis. The pyrrolic N_4_ coordination environment exhibits superior OER performance compared to its pyridinic counterpart, achieving an overpotential of 351 mV at 10 mA cm^−2^ in acidic media. Through combined DFT calculations and operando spectroscopic studies, we established that the pyrrolic coordination environment promotes enhanced OH^−^ adsorption and OER kinetics through its unique electronic structure and geometric flexibility. Notably, we discovered a multi‐layered protective mechanism in Pyrrolic CoN_4_‐CG that enables exceptional stability during acidic OER operation. This unprecedented stability originates from the higher defect formation energy of Co sites and the strategic distribution of sacrificial nitrogen species in the graphene network. The abundant pyrrolic and pyridinic nitrogen species serve as protective barriers, undergoing preferential dissolution while preserving the integrity of the catalytic Co‐N_4_ active sites. These findings not only demonstrate a high‐performance acidic OER catalyst but also provide fundamental design principles for developing stable single‐atom catalysts in challenging electrochemical applications.

## Conflict of Interest

The authors declare no conflict of interest.

## Supporting information



Supporting Information

## Data Availability

The data that support the findings of this study are available from the corresponding author upon reasonable request.

## References

[1] K. Zhang , R. Zou , Small 2021, 17, 2100129. 10.1002/smll.202100129 34114334

[2] A. Wu , Y. Xie , H. Ma , C. Tian , Y. Gu , H. Yan , X. Zhang , G. Yang , H. Fu , Nano Energy 2018, 44, 353.

[3] T. Shinagawa , M. T. K. Ng , K. Takanabe , Angew. Chem. 2017, 129, 5143. 10.1002/anie.201701642 28345220

[4] Y. Shi , Y. Yu , Y. Liang , Y. Du , B. Zhang , Angew. Chem., Int. Ed. 2019, 58, 3769. 10.1002/anie.201811241 30549367

[5] I. Katsounaros , S. Cherevko , A. R. Zeradjanin , K. J. Mayrhofer , Angew. Chem., Int. Ed. 2014, 53, 102. 10.1002/anie.201306588 24339359

[6] J. Song , C. Wei , Z.‐F. Huang , C. Liu , L. Zeng , X. Wang , Z. J. Xu , Chem. Soc. Rev. 2020, 49, 2196. 32133479 10.1039/c9cs00607a

[7] M. Tahir , L. Pan , F. Idrees , X. Zhang , L. Wang , J.‐J. Zou , Z. L. Wang , Nano Energy 2017, 37, 136.

[8] J. Shan , C. Ye , S. Chen , T. Sun , Y. Jiao , L. Liu , C. Zhu , L. Song , Y. Han , M. Jaroniec , J. Am. Chem. Soc. 2021, 143, 5201. 33764061 10.1021/jacs.1c01525

[9] T. Wu , S. Sun , J. Song , S. Xi , Y. Du , B. Chen , W. A. Sasangka , H. Liao , C. L. Gan , G. G. Scherer , Nat. Catal. 2019, 2, 763.

[10] C. Zhu , S. Fu , Q. Shi , D. Du , Y. Lin , Angew. Chem., Int. Ed. 2017, 56, 13944. 10.1002/anie.201703864 28544221

[11] Y. Zhang , X. Zhu , G. Zhang , P. Shi , A.‐L. Wang , J. Mater. Chem. A 2021, 9, 5890

[12] K. Maiti , S. Maiti , M. T. Curnan , H. J. Kim , J. W. Han , Adv. Energy Mater. 2021, 11, 2101670.

[13] J.‐F. Sun , Q.‐Q. Xu , J.‐L. Qi , D. Zhou , H.‐Y. Zhu , J.‐Z. Yin , ACS Sustainable Chem. Eng. 2020, 8, 14630.

[14] S. Xiao , D. Zhang , D. Pan , W. Zhu , P. Liu , Y. Cai , G. Li , H. Li , Nat. Commun. 2019, 10, 1570. 30952853 10.1038/s41467-019-09509-y PMC6450964

[15] H. Xiang , W. Feng , Y. Chen , Adv. Mater. 2020, 32, 1905994. 10.1002/adma.201905994 31930751

[16] X.‐P. Zhang , A. Chandra , Y.‐M. Lee , R. Cao , K. Ray , W. Nam , Chem. Soc. Rev. 2021, 50, 4804. 33657202 10.1039/d0cs01456g

[17] J. Jing , J. Yang , Z. Zhang , Y. Zhu , Adv. Energy Mater. 2021, 11, 2101392.

[18] H. Lv , X. P. Zhang , K. Guo , J. Han , H. Guo , H. Lei , X. Li , W. Zhang , U. P. Apfel , R. Cao , Angew. Chem., Int. Ed. 2023, 62, 202305938. 10.1002/anie.202305938 37550259

[19] J. Limburg , J. S. Vrettos , L. M. Liable‐Sands , A. L. Rheingold , R. H. Crabtree , G. W. Brudvig , Science 1999, 283, 1524. 10066173 10.1126/science.283.5407.1524

[20] L. Xie , X. P. Zhang , B. Zhao , P. Li , J. Qi , X. Guo , B. Wang , H. Lei , W. Zhang , U. P. Apfel , Angew. Chem. 2021, 133, 7654. 10.1002/anie.202015478 33462971

[21] R. Zhang , J. J. Warren , ChemSusChem 2021, 14, 293. 33064354 10.1002/cssc.202001914

[22] P. Gotico , Z. Halime , A. Aukauloo , Dalton Trans. 2020, 49, 2381. 32040100 10.1039/c9dt04709c

[23] G.‐G. Luo , H.‐L. Zhang , Y.‐W. Tao , Q.‐Y. Wu , D. Tian , Q. Zhang , Inorg. Chem. Front. 2019, 6, 343.

[24] S. Nam , M. Mahato , K. Matthews , R. W. Lord , Y. Lee , P. Thangasamy , C. W. Ahn , Y. Gogotsi , I. K. Oh , Adv. Funct. Mater. 2023, 33, 2210702.

[25] Z. Ren , B. Zhao , J. Xie , Small 2023, 19, 2301818. 10.1002/smll.202301818 37010014

[26] C. H. Lee , D. K. Dogutan , D. G. Nocera , J. Am. Chem. Soc. 2011, 133, 8775. 21557608 10.1021/ja202136y

[27] Y. Chen , S. Ji , C. Chen , Q. Peng , D. Wang , Y. Li , Joule 2018, 2, 1242.

[28] D. Yang , B. Ni , X. Wang , Adv. Energy Mater. 2020, 10, 2001142.

[29] H. Tian , A. Song , P. Zhang , K. Sun , J. Wang , B. Sun , Q. Fan , G. Shao , C. Chen , H. Liu , Adv. Mater. 2023, 35, 2210714. 10.1002/adma.202210714 36630970

[30] Y. Sun , S. Sun , H. Yang , S. Xi , J. Gracia , Z. J. Xu , Adv. Mater. 2020, 32, 2003297. 10.1002/adma.202003297 32776367

[31] W. Song , C. Xiao , J. Ding , Z. Huang , X. Yang , T. Zhang , D. Mitlin , W. Hu , Adv. Mater. 2023, 36, 2301477. 10.1002/adma.202301477 37078970

[32] X. Yao , Y. Zhu , T. Xia , Z. Han , C. Du , L. Yang , J. Tian , X. Ma , J. Hou , C. Cao , Small 2023, 19, 2301075. 10.1002/smll.202301075 36978240

[33] S. W. Han , J. Bang , S. H. Ko , R. Ryoo , J. Mater. Chem. A 2019, 7, 8353.

[34] Y. Kwon , K. Kim , R. Ryoo , RSC Adv. 2016, 6, 43091.

[35] S. Ye , F. Luo , Q. Zhang , P. Zhang , T. Xu , Q. Wang , D. He , L. Guo , Y. Zhang , C. He , Energy Environ. Sci. 2019, 12, 1000.

[36] J. H. Kim , D. Shin , J. Lee , D. S. Baek , T. J. Shin , Y.‐T. Kim , H. Y. Jeong , J. H. Kwak , H. Kim , S. H. Joo , ACS Nano 2020, 14, 1990. 31999424 10.1021/acsnano.9b08494

[37] X. Zhang , Z. Wu , X. Zhang , L. Li , Y. Li , H. Xu , X. Li , X. Yu , Z. Zhang , Y. Liang , Nat. Commun. 2017, 8, 14675. 28272403 10.1038/ncomms14675 PMC5344970

[38] J. Zhang , H. Chen , S. Liu , L.‐D. Wang , X.‐F. Zhang , J.‐X. Wu , L.‐H. Yu , X.‐H. Zhang , S. Zhong , Z.‐Y. Du , J. Am. Chem. Soc. 2023, 145, 20000. 37610355 10.1021/jacs.3c06665

[39] Q. Chang , Y. Liu , J.‐H. Lee , D. Ologunagba , S. Hwang , Z. Xie , S. Kattel , J. H. Lee , J. G. Chen , J. Am. Chem. Soc. 2022, 144, 16131. 36007154 10.1021/jacs.2c06953

[40] W. Zhang , L. Wang , L. H. Zhang , D. Chen , Y. Zhang , D. Yang , N. Yan , F. Yu , ChemSusChem 2022, 15, 202200195. 10.1002/cssc.202200195 PMC9311226 35244341

[41] W. Fan , Z. Duan , W. Liu , R. Mehmood , J. Qu , Y. Cao , X. Guo , J. Zhong , F. Zhang , Nat. Commun. 2023, 14, 1426. 36918545 10.1038/s41467-023-37066-y PMC10014850

[42] N. Zhang , T. Zhou , M. Chen , H. Feng , R. Yuan , W. Yan , Y. Tian , X. Wu , W. Chu , C. Wu , Energy Environ. Sci. 2020, 13, 111.

[43] L. Yan , L. Xie , X. L. Wu , M. Qian , J. Chen , Y. Zhong , Y. Hu , Carbon Energy 2021, 3, 856.

[44] H. Xu , D. Cheng , D. Cao , X. C. Zeng , Nat. Catal. 2018, 1, 339.

[45] J. K. Nørskov , J. Rossmeisl , A. Logadottir , L. Lindqvist , J. R. Kitchin , T. Bligaard , H. Jonsson , J. Phys. Chem. B 2004, 108, 17886. 39682080 10.1021/jp047349j

[46] T.‐H. Kim , E. K. Jeon , Y. Ko , B. Y. Jang , B.‐S. Kim , H.‐K. Song , J. Mater. Chem. A 2014, 2, 7600.

[47] H. Asgar , K. Deen , U. Riaz , Z. U. Rahman , U. H. Shah , W. Haider , Mater. Chem. Phys. 2018, 206, 7.

[48] Y. Han , Y.‐G. Wang , W. Chen , R. Xu , L. Zheng , J. Zhang , J. Luo , R.‐A. Shen , Y. Zhu , W.‐C. Cheong , J. Am. Chem. Soc. 2017, 139, 17269. 29108411 10.1021/jacs.7b10194

[49] H. Zhang , W. Zhou , T. Chen , B. Y. Guan , Z. Li , X. W. D. Lou , Energy Environ. Sci. 2018, 11, 1980.

[50] M. D. Hall , G. J. Foran , M. Zhang , P. J. Beale , T. W. Hambley , J. Am. Chem. Soc. 2003, 125, 7524. 12812486 10.1021/ja0354770

[51] M. Cheng , H. Zhao , Z. Zhao , J. Wang , L. Cao , H. Zhang , X. Duan , C. Wang , J. Wang , J. Wang , Green Chem. 2018, 20, 4675.

[52] P. Chen , N. Zhang , S. Wang , T. Zhou , Y. Tong , C. Ao , W. Yan , L. Zhang , W. Chu , C. Wu , Proc. Natl. Acad. Sci. USA 2019, 116, 6635. 30872473 10.1073/pnas.1817881116 PMC6452708

[53] I. Y. Kim , S. Kim , X. Jin , S. Premkumar , G. Chandra , N. S. Lee , G. P. Mane , S. J. Hwang , S. Umapathy , A. Vinu , Angew. Chem. 2018, 130, 17381. 10.1002/anie.201811061 30407712

[54] I. Matanovic , K. Artyushkova , M. B. Strand , M. J. Dzara , S. Pylypenko , P. Atanassov , J. Phys. Chem. C. 2016, 120, 29225.

[55] T.‐N. Tran , C.‐H. Shin , B.‐J. Lee , J. S. Samdani , J.‐D. Park , T.‐H. Kang , J.‐S. Yu , Catal. Sci. Technol. 2018, 8, 5368.

[56] J. Masa , W. Xia , M. Muhler , W. Schuhmann , Angew. Chem., Int. Ed. 2015, 54, 10102. 10.1002/anie.201500569 26136398

[57] M. Liu , J. Zhang , H. Su , Y. Jiang , W. Zhou , C. Yang , S. Bo , J. Pan , Q. Liu , Nat. Commun. 2024, 15, 1675. 38396104 10.1038/s41467-024-45990-w PMC10891135

[58] H. Su , M. A. Soldatov , V. Roldugin , Q. Liu , EScience 2022, 2, 102.

[59] S. A. Baudron , Coord. Chem. Rev. 2019, 380, 318.

