# Supplementary material for: Ligand Engineering of Co‐N4 Single‐Atom Catalysts for Highly‐Active and Stable Acidic Oxygen Evolution
Source: Adv Sci (Weinh). 2025 Apr 30;12(27):2502230. doi: 10.1002/advs.202502230 (PMC12279189; doi:10.1002/advs.202502230)
Supplement: Supplementary file 1 — Supporting Information [file ADVS-12-2502230-s001.docx]

**Supporting Information**

**Ligand Engineering of Co-N_4_ Single-Atom Catalysts for Highly-Active and Stable Acidic Oxygen Evolution**

Taeyoung Jeong, Kiwon Kim, Byung-Hyun Kim, Sang-Il Choi, Chang Hyuck Choi*, Joonhee Kang* and Myeongjin Kim*

T. Jeong, K.Kim, Prof. M. Kim

School of Energy Engineering, Kyungpook National University, 80 Daehak-ro, Bukgu, Daegu 41566, Republic of Korea

E-mail: myeongjinkim@knu.ac.kr

Prof. B.-H. Kim

Department of Chemical and Molecular Engineering, Hanyang University ERICA, 55 Hanyangdaehak-ro, Sangnok-gu, Ansan-si, Gyeonggi-do 15588, Republic of Korea

Department of Applied Chemistry, Center for Bionano Intelligence Education and Research, Hanyang University ERICA, 55 Hanyangdaehak-ro, Sangnok-gu, Ansan-si, Gyeonggi-do 15588, Republic of Korea

Prof. S.-I. Choi

Department of Chemistry and Green-Nano Materials Research Center, Kyungpook National University, 80 Daehak-ro, Bukgu, Daegu 41566, Republic of Korea

Prof. C. H. Choi

Department of Chemistry, Pohang University of Science and Technology (POSTECH), 77 Cheongam-ro, Nam-gu, Pohang 37673, Republic of Korea

Institute for Convergence Research and Education in Advanced Technology (I-CREATE), Yonsei University, 50 Yonsei-ro, Seodaemun-gu, Seoul 03722, Republic of Korea

E-mail: chchoi@postech.ac.kr

Prof. J. Kang

Department of Nano Fusion Technology, Pusan National University, 2 Busandaehak-ro 63beon-gil, Geumjeong-gu, Busan 46241, Republic of Korea

Department of Nanoenergy Engineering, Pusan National University, 2 Busandaehak-ro 63beon-gil, Geumjeong-gu, Busan 46241, Republic of Korea

E-mail: j.kang@pusan.ac.kr

**Methods**

**Preparation of crumpled graphene**. Firstly, the graphene oxide (GO) was prepared through modified Hummer’s method. The 3 g of graphite powder was dissolved in a mixture containing 360 mL of concentrated H_2_SO_4_ and 40 mL of concentrated H_3_PO_4_, followed by stirring in an ice bath for 4 hours. Then, 18 g of KMnO_4_ at room temperature in an ice bath was slowly added to the uniformly dispersed graphite powder over 1 hour. After stirring the mixture at 50 °C for 12 hours in an oil bath, the obtained mixture was poured into 400 mL of deionized water (DI) containing 3 mL of H_2_O_2_. The mixture was thoroughly cleaned and purified with HCl and DI water, respectively, and then graphite oxide was collected. To obtain GO by exfoliation, graphite oxide was dispersed ultrasonically in deionized water. After drying in a vacuum oven at 80 °C for 2 days, the GO powder was collected. After concentration control of GO (5 mg mL^-1^) in deionized water, the GO solution was sprayed into a tube furnace preheated to 100 °C through a mini spray dryer with an Ar gas flow (10 L min^-1^). The water in the sprayed GO droplets evaporates rapidly during flight in the chamber. Rapid evaporation shrinks the droplets and compresses them into crumple graphene oxide (CGO). The CGO powder was annealed in an argon gas atmosphere in a quartz tubular furnace at 1100 °C for 2 hours. After thermal annealing process, CGO was reduced to the target material, crumpled graphene (CG).

**Preparation of Pyrrolic CoN_4_-CG and Pyridinic CoN_4_-CG**. The 1.38 ml of 0.6 M CoCl_2_ aqueous solution was added dropwise to a mixture containing 200 ml of 0.5 M Hydrochloric acid (HCl) solution and 2 ml of aniline. The resulting mixture was stirred at 4°C for 1 hour. For polymerization of aniline, 20 ml of 1.1 M (NH_4_)_2_S_2_O_8_ aqueous solution was added dropwise, and then 400mg of CG was added into the solution. The mixture was stirred at room temperature for 48 hours and the required precursor was gathered. The precursor was vacuum dried at 60°C, and then pyrolyzed at 900°C for 1 hour with a stream of NH_3_ gas to facilitate the development of Co-pyrrolic N coordination environment. Thereafter, the obtained product was subjected to a leaching process with 2 M H_2_SO_4_ at 60 °C for 12 hours. The product was washed meticulously with DI water and then vacuum dried at 60 °C. The resulting product was gathered and denoted as Pyrrolic CoN_4_-CG. The synthesis process of Pyridinic CoN_4_-CG is almost identical to that of Pyrrolic CoN_4_-CG synthesis, except that it is pyrolyzed in argon gas atmosphere instead of NH_3_ gas flow. The synthesis process of Pyrrolic NCG and Pyridinic NCG is almost identical to that of Pyrrolic CoN_4_-CG and Pyridinic CoN_4_-CG, except that the synthesis is performed without a metal precursor.

**Preparation of working electrode**. The 7.5 mg of Pyrrolic CoN_4_-CG, Pyridinic CoN_4_-CG, CG and IrO_2_ (FuelCellStore, 12 nm of average particle size, 99.9% trace metals basis) were blended with 0.86 mL of ethanol, 0.1 mL of DI water, and 0.038mL of 5 wt% Nafion (Sigma-Aldrich, 5 wt% in isopropanol). The prepared slurry was ultrasonicated for 30 minutes to produce a catalyst ink. Then, 4 μL of catalyst ink was dropped onto a glassy carbon electrode (PINE, 0.196 cm^2^, 5 mm diameter), and the resulting electrode was dried at room temperature. The loading mass of catalyst on the glassy carbon electrode for Pyrrolic CoN_4_-CG, Pyridinic CoN_4_-CG, CG, and IrO_2_ is 0.213, 0.211, 0.209 and 0.208 mg_catalyst_ cm^-2^, respectively.

**Electrochemcial measurements.** To investigate the catalytic activities of the oxygen evolution reaction (OER) in 0.5 M H_2_SO_4_ aqueous electrolyte, the rotating disk electrode (RDE) technique was performed by a three-electrode cell setup comprising a glassy carbon rotating disk electrode, Pt wire, and Ag/AgCl (sat. KCl) as working, counter, and reference electrode, respectively. RHE calibration of the Ag/AgCl (sat. KCl) reference electrode was conducted in 0.5 M H_2_SO_4_. Prior to calibration, 0.5M H_2_SO_4_ aqueous electrolyte was purged with ultra-high purity hydrogen gas (99.999%) for 30 minutes to be saturated with hydrogen. The calibration was performed in H_2_-saturated 0.5 M H_2_SO_4_ electrolyte using Pt RDE as the working electrode. Forward and reverse CV scans were run at a scan rate of 1 mV s^-1^ and the thermodynamic potential for the hydrogen electrode reaction was decided via the average of the two potentials where the current intersects zero. Prior to confirming the OER activity of the as-prepared electrocatalysts through RDE experiments, the 0.5 M H_2_SO_4_ aqueous electrolyte was purged with ultra high purity oxygen gas (99.995%) for 30 minutes to be saturated with oxygen. Polarization curves were collected at a 1600 rpm rotation rate and a 5 mV s^-1^ scan rate in 0.5 M H_2_SO_4_ aqueous electrolyte saturated with oxygen. The iR-compensation was performed for all potential values to correct the Ohmic resistance of the solution, according to the following equation:

𝐸_iR-corrected_=𝐸–𝑖𝑅 (1)

The catalyst stabibility test was performed by chronoamperometry measurement at a constant applied potential over 20 hours.

**Characterization methods.** The morphologies of the samples were analyzed using field-emission scanning electron microscopy (FE-SEM, SIGMA, Carl Zeiss) and high-resolution transmission electron microscopy (HR-TEM, JEM-2100F, JEOL) at 200 kV. High-angle annular dark-field scanning transmission electron microscopy (HAADF-STEM) and corresponding energy dispersive spectroscopy (EDS) mapping analyses are executed on JEOL JEM-ARF200F TEM/STEM with a spherical aberration corrector. Elemental analyses are conducted on FlashSmart™ Elemental Analyzer in CHN Mode. X-ray diffraction (XRD, New D8-Advance, Bruker-AXS) patterns were collected at a scan rate of 1 s^-1^ within the 2θ range of 10°–80° and using Cu K_α1_ radiation (0.154056 nm). X-ray photoelectron spectroscopy (XPS) analysis was performed on a PHI 5000 VersaProbe II (Japan) with a monochromatic Al Kα (hv = 1486.69 eV) X-ray source. X-ray absorption spectroscopy (XAS) analyses were conducted on the BL7D beam line and Soft X-ray absorption spectroscopy (sXAS) analysis was conducted on the BL8A beam line at the Pohang Light Source (PLS, Korea) of Pohang Accelerator Laboratory (PAL). For XAS, the incident beam was collimated by a Ru-coated mirror at 2.8 mrad and monochromatized using a channel-cut Si (1 1 1) monochromator under the operation in top-up mode under a ring current of 300 mA at 3.0 GeV. The acquired extended X-ray absorption fine structure (EXAFS) data were processed according to standard procedures using the ATHENA module implemented in the IFEFFIT software packages. The EXAFS spectra were obtained by subtracting the post-edge background from the overall absorption and then normalizing it with respect to the edge-jump step. Subsequently, the χ(k) data in k-space were Fourier-transformed to real (R) space using a Hanning window to separate the EXAFS contributions from different coordination shells. *Operando* XAS analysis was conducted using an in-house developed three-electrode configuration consisting of an as-prepared electrocatalyst-based air electrode, Ag/AgCl (KCl Sat.) and Pt wire as working, reference, and counter electrode, respectively, in 0.5 M H_2_SO_4_ aqueous electrolyte. Briefly, X-ray photons entered the air electrode through the backside of it, and fluorescent photons exiting the electrode were detected at 90 degrees relative to the incident beam. For the electrocatalyst-based air electrode fabrication, glassy carbon plates were used as working electrodes, serving as substrates for the catalysts. The as-prepared catalysts ink was dropped on the plates and fully dried. Then the working electrode was attached to the cells with fast-curing epoxy (Devcon). Conductive Cu tape was linked to the backside of the working electrode to make the electrical connection. *Operando* XAS measurements were carried out in top-up mode under a ring current of 300 mA at 3.0 GeV and recorded at open circuit potential (OCP), 1.48, 1.50, 1.52, 1.54, 1.56 and 1.58 V vs. RHE, and the each applied potential was stabilized for 5 min before XAS experiments, respectively. Nitrogen sorption analysis was carried out using an ASAP 2020 accelerated surface area and porosimetry instrument (Micromeritics), equipped with an automated surface area at 77 K, using Brunauer–Emmett–Teller (BET) calculations for the surface area. The pore-size distribution plots were recorded from the desorption branch of the isotherms based on the nonlocal density functional theory (NLDFT). The weight percentage of Co in Co containing single atom catalysts were investigated by inductively coupled plasma atomic emission spectroscopy (ICP-AES) analysis using a TJA IRIS (HR) spectrometer. The mass of Co dissolution during the CP tests was determined by inductively coupled plasma mass spectrometer (ICP-MS) analysis using Perkin Elmer (NexION 2000).

**Computational methods**. The electronic structure calculations were performed using the Vienna Ab Initio Simulation Package (VASP).^[1-4]^ The generalized gradient approximation (GGA)^[5]^ with the Perdew–Burke–Ernzerhof functional was used to represent the exchange-correlation energy, and the projector augmented-wave pseudo potential was used to describe the core electrons.^[6]^ The cutoff energy for the plane-wave basis set was set to 500 eV. The energy and force convergence criterion of the model system was set to 10^–5^ eV and 0.02 eV/Å. A vacuum region of about 15 Å was used to prevent periodic interactions. The Brillouin zone was sampled with 3 × 3 × 1 Gamma-centered k-points. The DFT calculations incorporated the D3 dispersion correction scheme proposed by Grimme, with the zero-damping function.^[7]^ To accurately evaluate the *d* orbital states, the screened hybrid functionals as proposed by Heyd-Scuseria-Ernzerhof (HSE06) were used for density of states (DOS) calculations.^[8]^


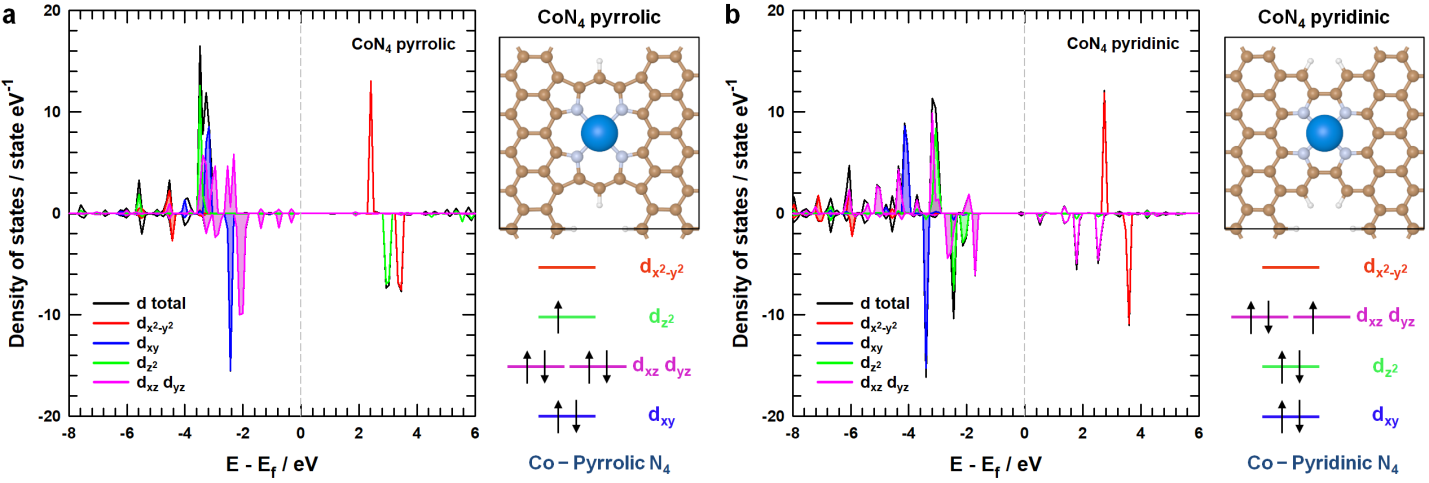


**Figure S1.** Electronic structure and spin state of Co^2+^ within pyridinic or pyrrolic N_4_ ligand structure. Calculated projected density of states (PDOS) and *d* orbital arrangement of Co^2+^ for (a) CoN_4_ pyrrolic and (b) CoN_4_ pyridinic model. Generally, the $d_{xz,}d_{yz}$ and $d_{z^{2}}$ states of transition metal with the square planar (D_4h_) symmetry are stabilized due to the absence of z-direction ligands. However, in the case of σ-donor ligands, the torus-shaped $d_{z^{2}}$ orbital participates in metal-ligand interactions in the xy plane, resulting in an unstable $d_{z^{2}}$ state like CoN_4_ pyrrolic (Figure S1a).^[9]^ Also, the charge density difference in Figure 1b shows that Co loses $d_{z^{2}}$ electrons in the pyrrolic system. Conversely, as in CoN_4_ pyridinic, e_g_ ($d_{xz}$ and $d_{yz}$) orbitals become unstable when the N $p_{z}$ orbital repulsion becomes stronger as the Co-N bond length decreases. Thus, $d_{z^{2}}$/e_g_ orbital reversal occurs when CoN_4_ goes from pyrrolic to pyridinic N_4_ ligand structure (Figure S1b), but the spin state is the same (S = 1/2).


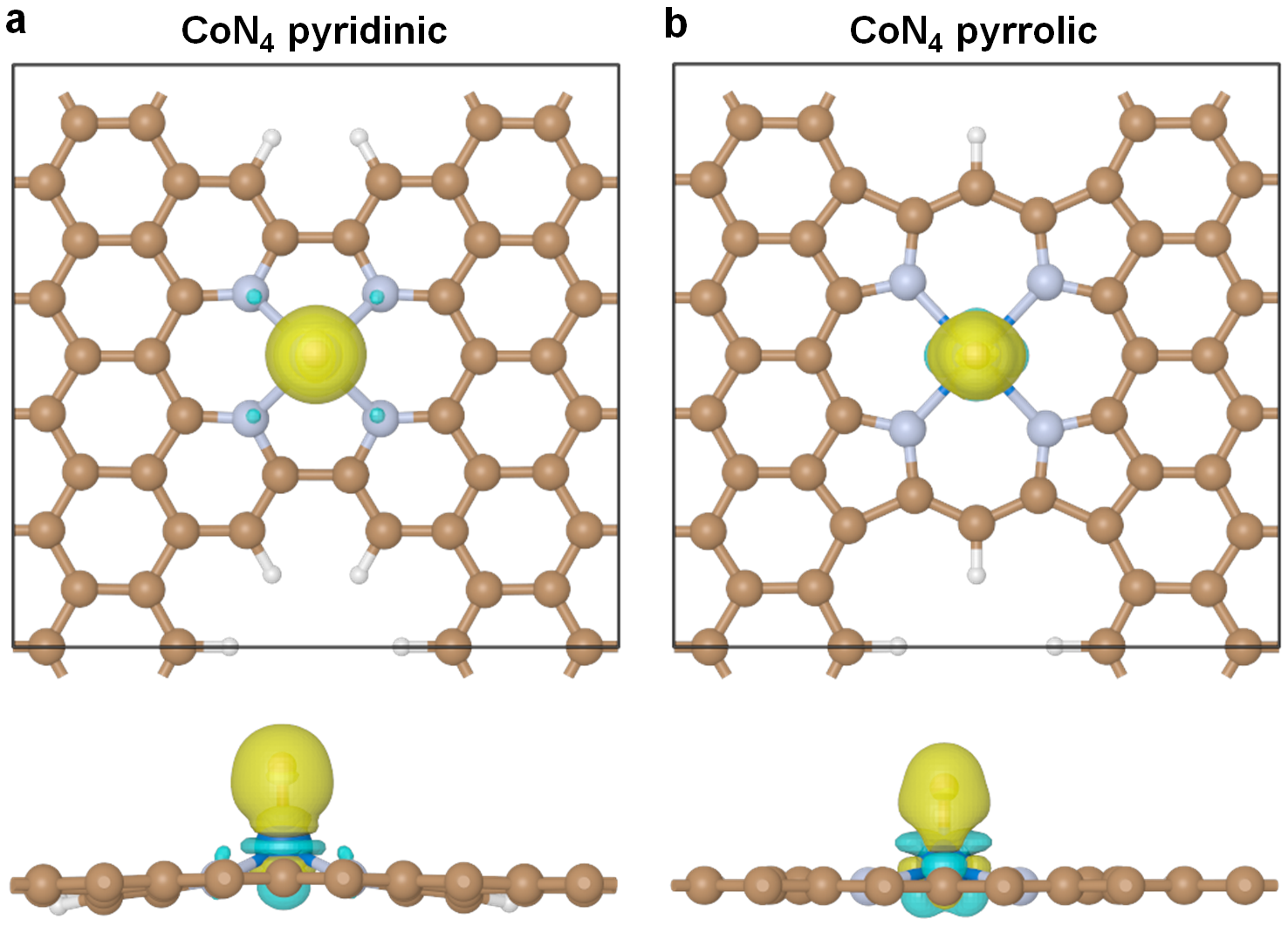


**Figure S2.** Charge density difference of oxygen-adsorbed (a) CoN_4_ pyridinic and (b) CoN_4_ pyrrolic model.


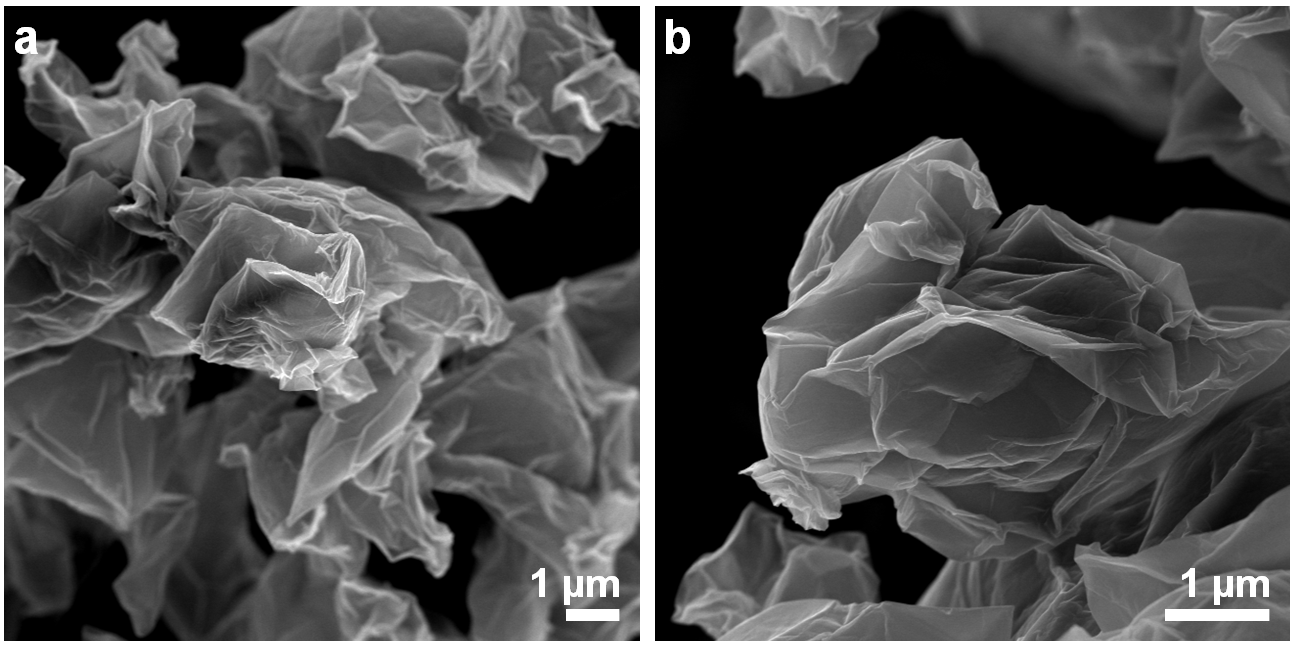


**Figure S3.** Field emission scanning electron microscopy (FE-SEM) images of Pyrrolic CoN_4_-CG. (a, b) FE-SEM images of Pyrrolic CoN_4_-CG at different magnifications.


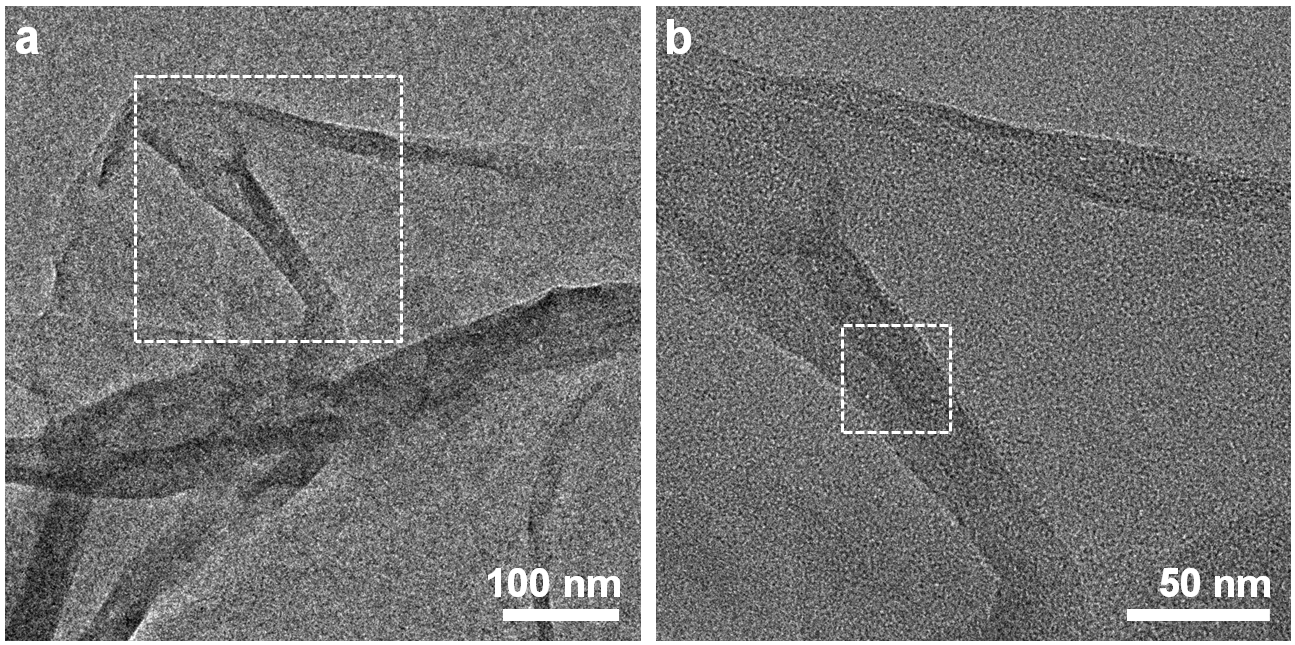


**Figure S4.** Field emission transmission electron microscopy (FE-TEM) images of Pyrrolic CoN_4_-CG. (a, b) FE-TEM images of Pyrrolic CoN_4_-CG at different magnifications showing the formation of ridge structure.


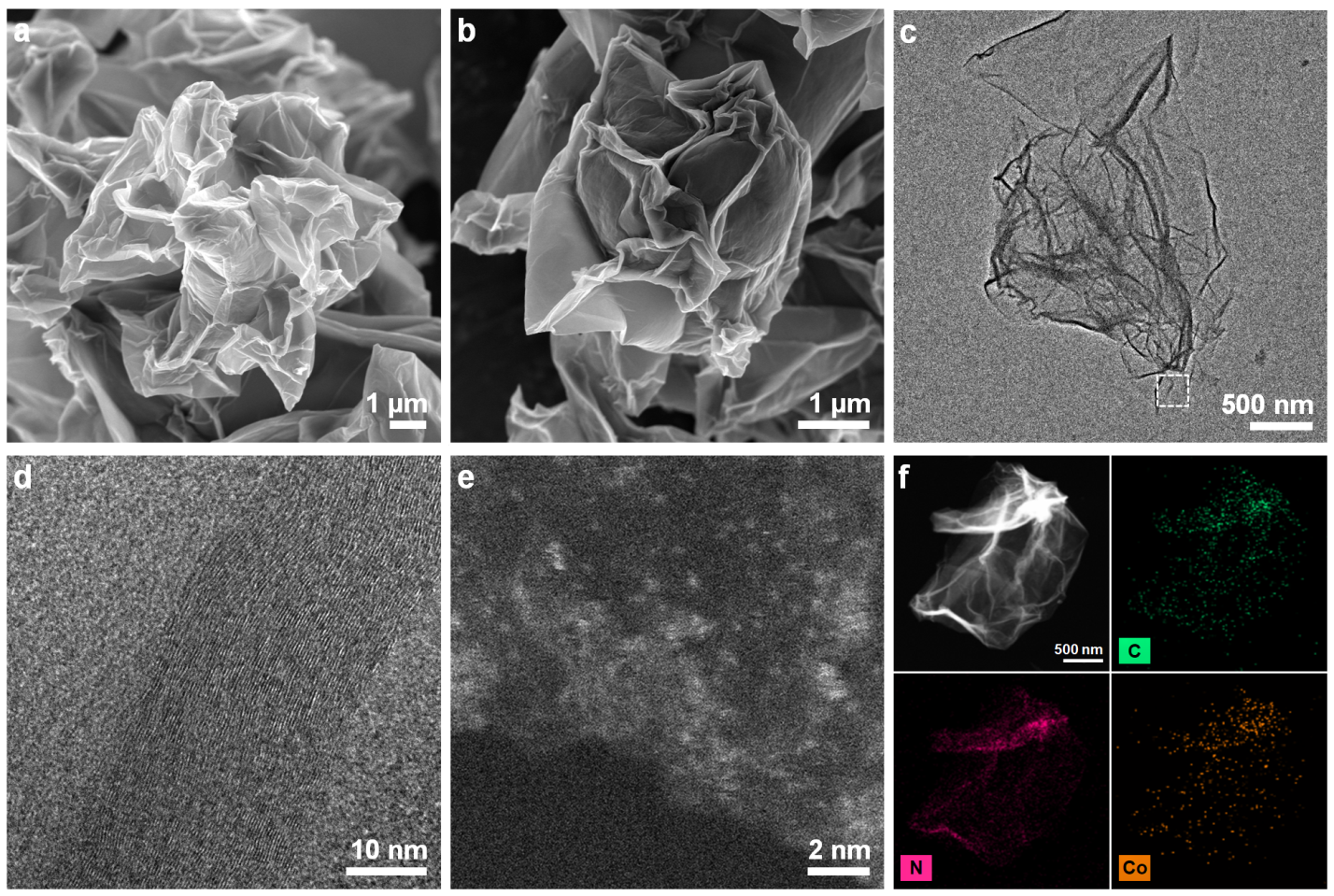


**Figure S5.** FE-SEM images and FE-TEM images of Pyridinic CoN_4_-CG. (a, b) FE-SEM images of Pyridinic CoN_4_-CG at different magnifications. (c, d) High-resolution transmission electron microscopy (HR-TEM) images of Pyridinic CoN_4_-CG at different magnifications. (e) High-angle annular dark field scanning transmission electron microscopy (HAADF-STEM) image of Pyridinic CoN_4_-CG. (f) Energy dispersive x-ray analysis (EDX) mapping of Pyridinic CoN_4_-CG.


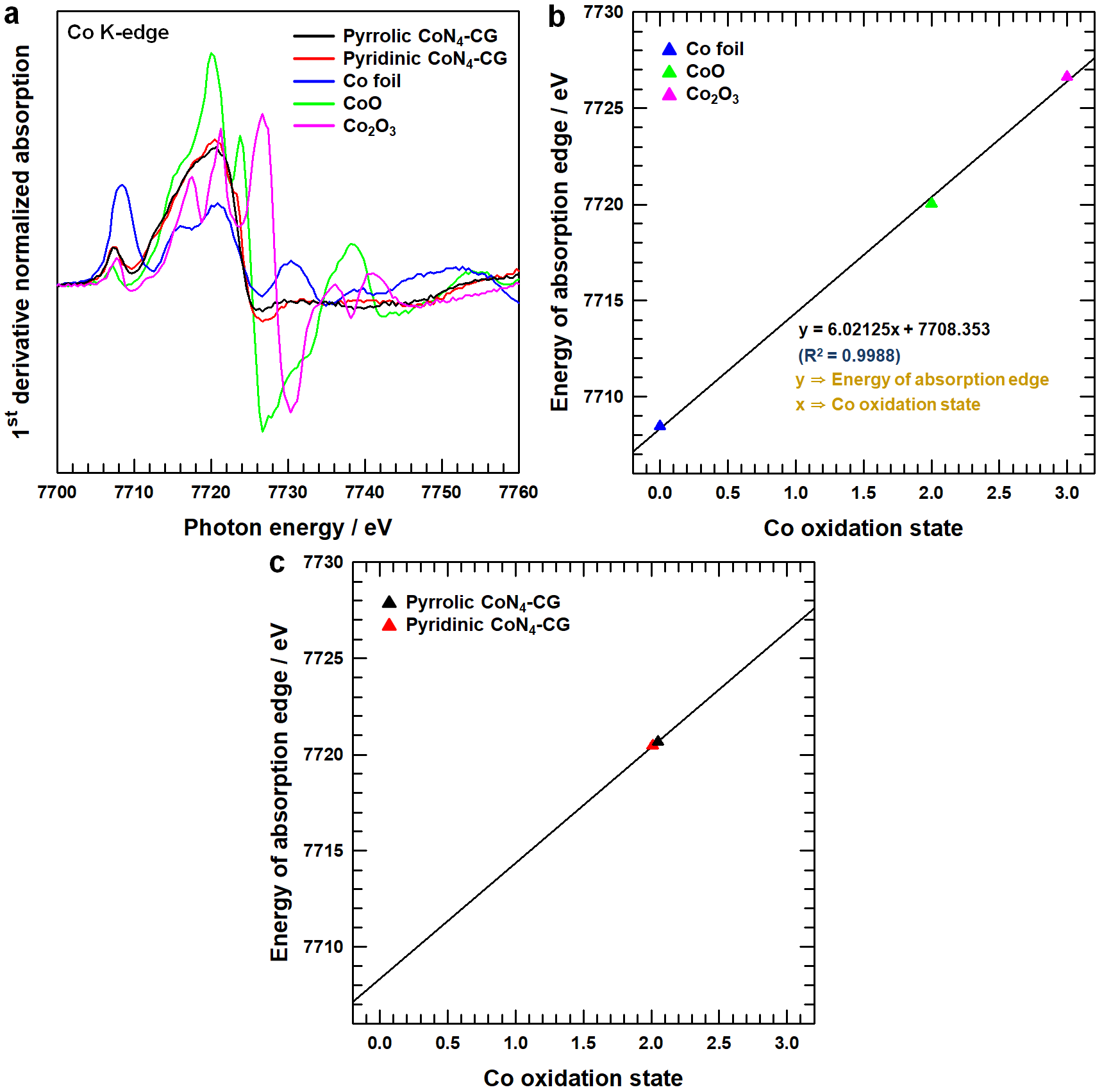


**Figure S6.** (a) First derivative of Co K-edge XANES spectra of Co foil, CoO, Co_2_O_3_, Pyrrolic CoN_4_-CG and Pyridinic CoN_4_-CG. (b) Plot of absorption edge position vs Co valence state for Co foil, CoO and Co_2_O_3_. (c). Plot of absorption edge position vs Co valence state for Pyrrolic CoN_4_-CG and Pyridinic CoN_4_-CG.

**
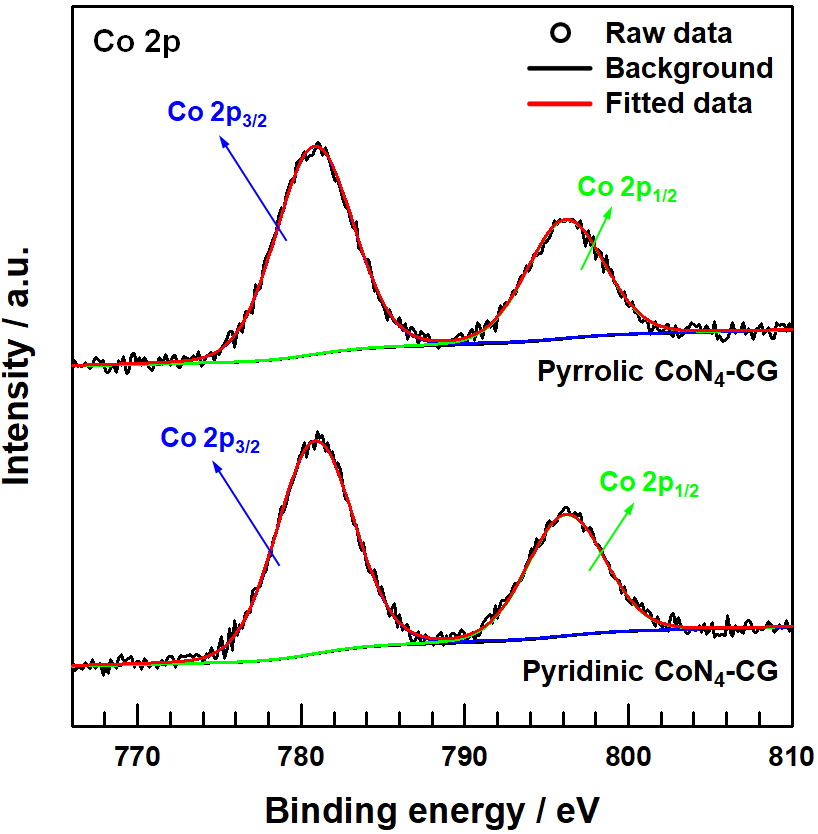
**

**Figure S7**. Co 2p X-ray photoelectron spectroscopy (XPS) spectra of Pyrrolic CoN_4_-CG and Pyridinic CoN_4_-CG.

| 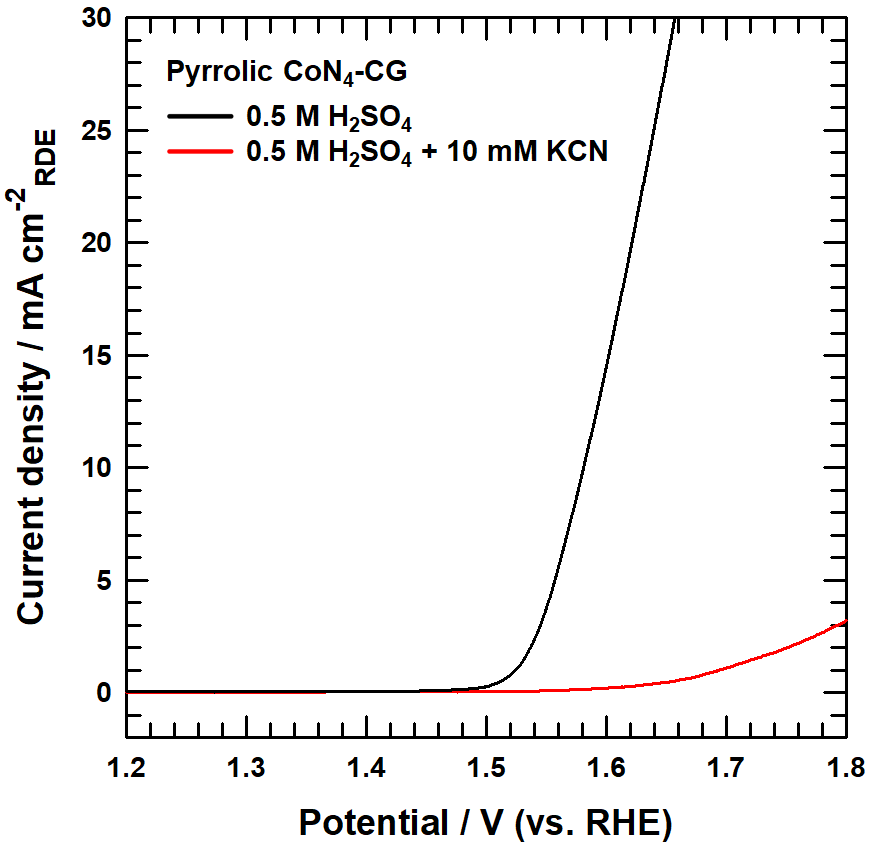 |
| --- |
| **Figure S8.** LSV polarization curves of Pyrrolic CoN_4_-CG before and after the addition of KCN in O_2_-saturated 0.5M H_2_SO_4_ electrolyte. With the introduction of 10 mM of KCN into the O_2_-saturated 0.5M H_2_SO_4_ electrolyte, the OER performance of Pyrrolic CoN_4_-CG plummeted after KCN poisoning. |

| 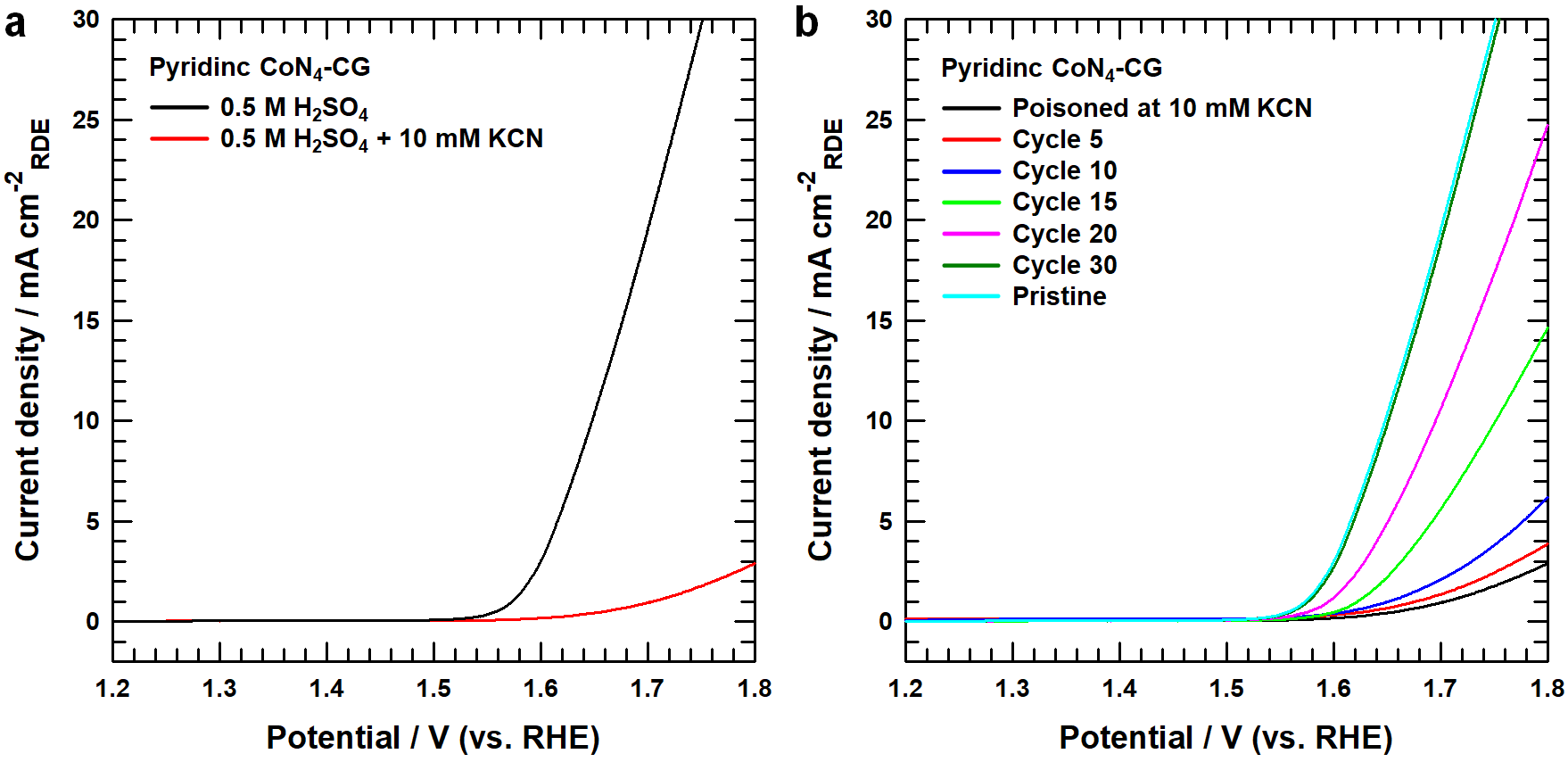 |
| --- |
| **Figure S9.** (a) LSV polarization curves of Pyridinic CoN_4_-CG before and after the addition of KCN in O_2_-saturated 0.5M H_2_SO_4_ electrolyte. With the introduction of 10 mM of KCN into the O_2_-saturated 0.5M H_2_SO_4_ electrolyte, the OER performance of Pyridinic CoN_4_-CG plummeted after KCN poisoning. (b) LSV polarization curves of KCN-poisoned Pyridinic CoN_4_-CG in O_2_-saturated 0.5M H_2_SO_4_ electrolyte. After the poisoned Pyridinic CoN_4_-CG was rinsed several times with water and remeasured in O_2_-saturated 0.5M H_2_SO_4_ electrolyte, the OER activity of poisoned Pyridinic CoN_4_-CG gradually recovered, and reach to the level of the fresh catalyst after 30 cycles due to the sufficient dissolution of the CN^-^. The poisoning and recovery experiments clearly imply that isolated Co atomic sites were the origin of the OER catalytic activity of Pyridinic CoN_4_-CG. |


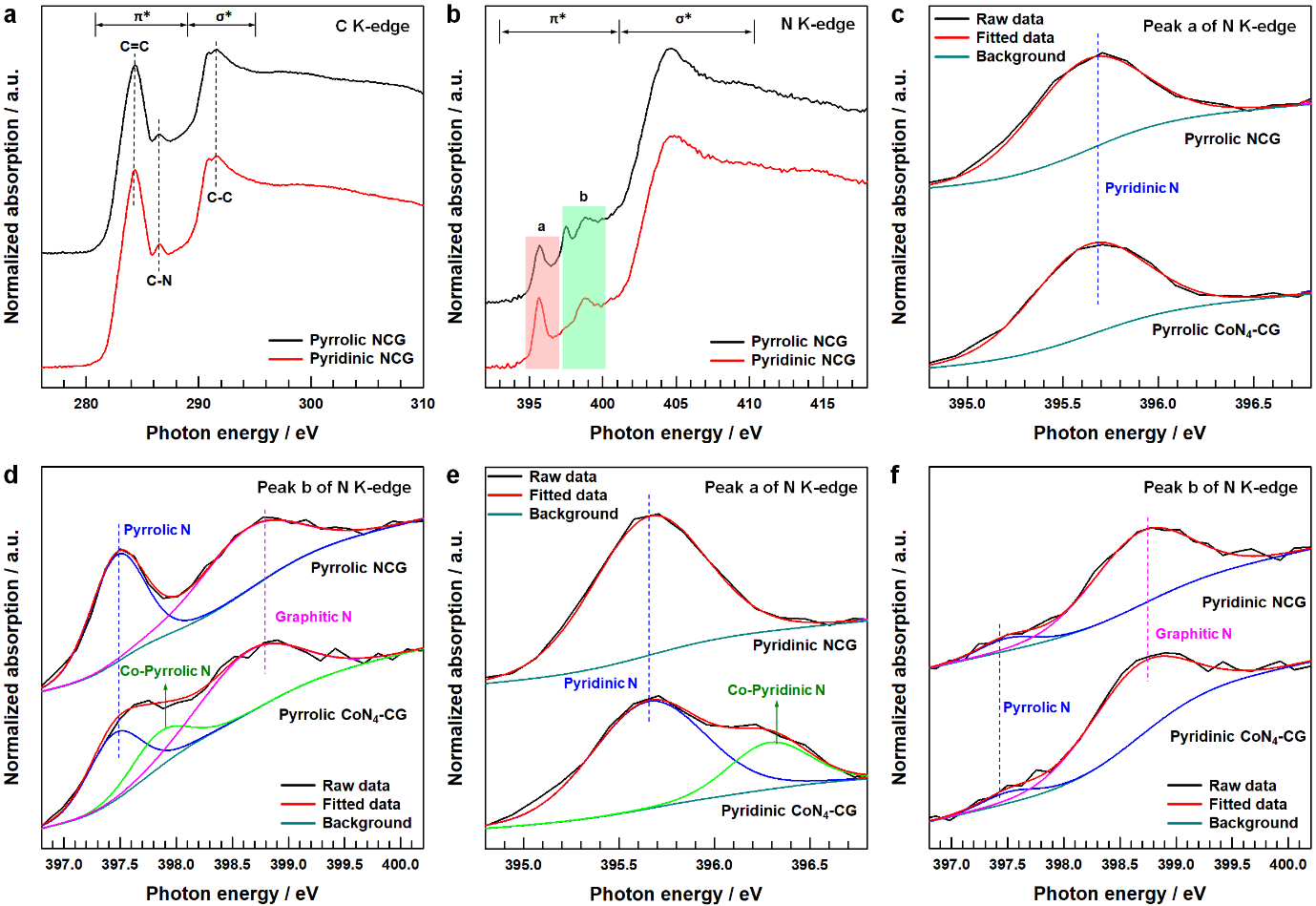


**Figure S10.** (a, b) C K-edge soft X-ray absorption near-edge spectroscopy (sXAS) spectra and N K-edge sXAS spectra of Pyrrolic NCG and Pyridinic NCG. (c, d) Peak a and Peak b of N K-edge sXAS spectra of Pyrrolic NCG and Pyrrolic CoN_4_-CG. (e, f) Peak a and Peak b of N K-edge sXAS spectra of Pyridinic NCG and Pyridinic CoN_4_-CG. Pyrrolic NCG and Pyridinic NCG show similar characteristics to the graphene, exhibiting π*_C=C_ peaks (around 284 eV) and σ*_C-C_ peaks (around 291.8 eV) in their C K edge sXAS spectra (Figure S10a). The C-N peak (286.4eV) appears in both pyrrolic NCG and pyridinic NCG, suggesting that N is successfully introduced into the graphene network. In the N K-edge spectrum (Figure S10b), all samples exhibit a π* peak (393.0-401.0 eV) and a σ* peak (401.0-410.0 eV). The fitted N K-edge spectrum of peak ‘a’ (Figure S10c) shows only pyridinic N (395.7 eV) for both Pyrrolic NCG and Pyrrolic CoN_4_-CG. In comparison, peak 'b' of Pyrrolic NCG (Figure S10d) exhibits pyrrolic N (397.5 eV) and graphitic N (398.8 eV) peaks, whereas Pyrrolic CoN_4_-CG shows all three peaks corresponding to pyrrolic N, graphitic N, and Co–pyrrolic N (398.0 eV). On the other hand, peak 'a' of Pyridinic NCG (Figure S10e) represents only the pyridinic N while Pyridinic CoN_4_-CG shows pyridinic N and Co-pyridinic N (398.9 eV). Both peak ‘b’ of Pyridinic NCG and Pyridinic CoN_4_-CG (Figure S10f) show two peaks of pyrrolic N and graphitic N.


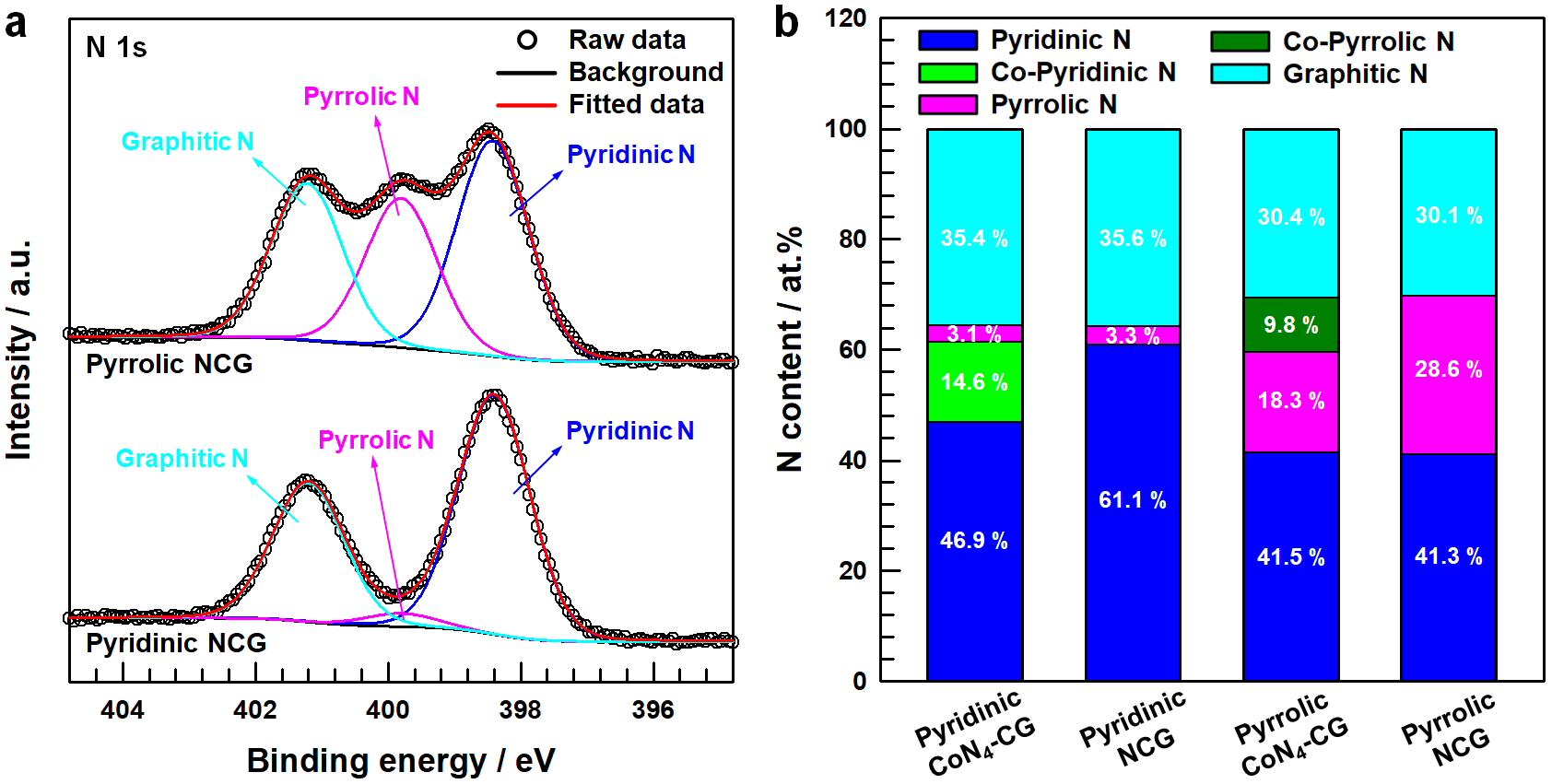


**Figure S11.** (a) N 1s X-ray photoelectron spectroscopy (XPS) spectra of Pyrrolic NCG and Pyridinic NCG. (b) N atomic ratio bar chart of Pyridinic CoN_4_-CG, Pyridinic NCG, Pyrrolic CoN_4_-CG, and Pyrrolic NCG. The combined atomic percentage of pyridinic N and Co-pyridinic N in Pyridinic CoN_4_-CG is comparable to that of pyridinic N in Pyridinic NCG. The same trend is also observed for the Pyrrolic samples. Therefore, Co-pyridinc N and Co-pyrrolic N sites in Pyridinic CoN_4_-CG and Pyrrolic CoN_4_-CG are completely converted to pyridinc N and pyrrolic N groups in Pyridinic NCG and Pyrrolic NCG, representing the successful fabrication of Co-free nitrogen doped CG.


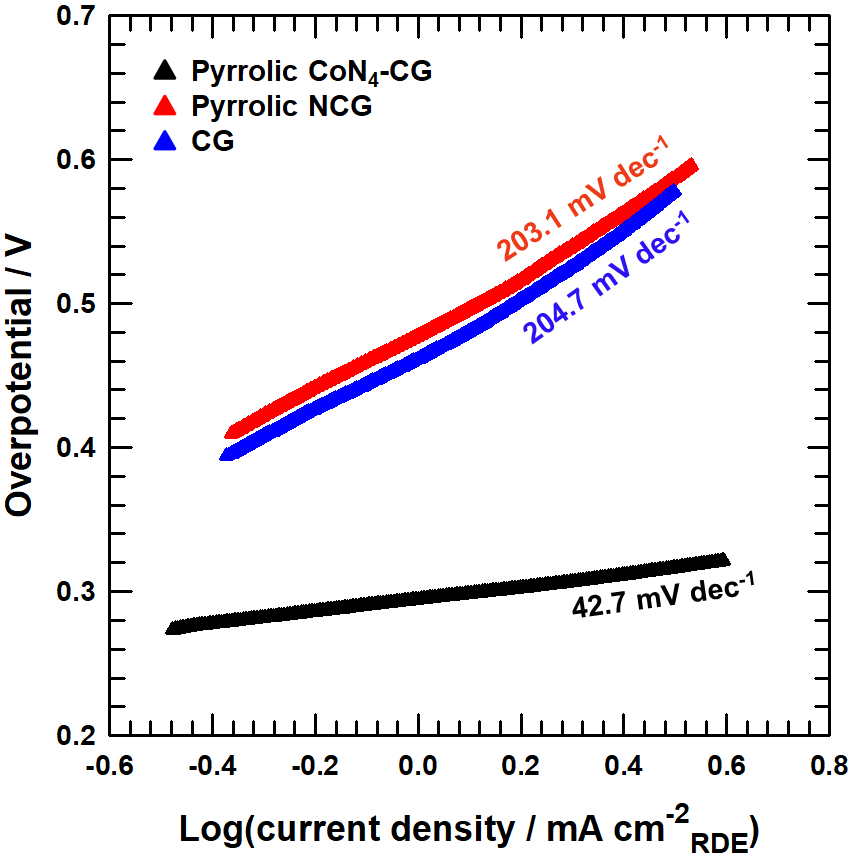


**Figure S12.** Tafel plots for Pyrrolic CoN_4_-CG, Pyrrolic NCG, and CG recorded at a scan rate of 5mV s^-1^ and a rotation rate of 1600 rpm in O_2_-saturated 0.5M H_2_SO_4_ electrolyte.


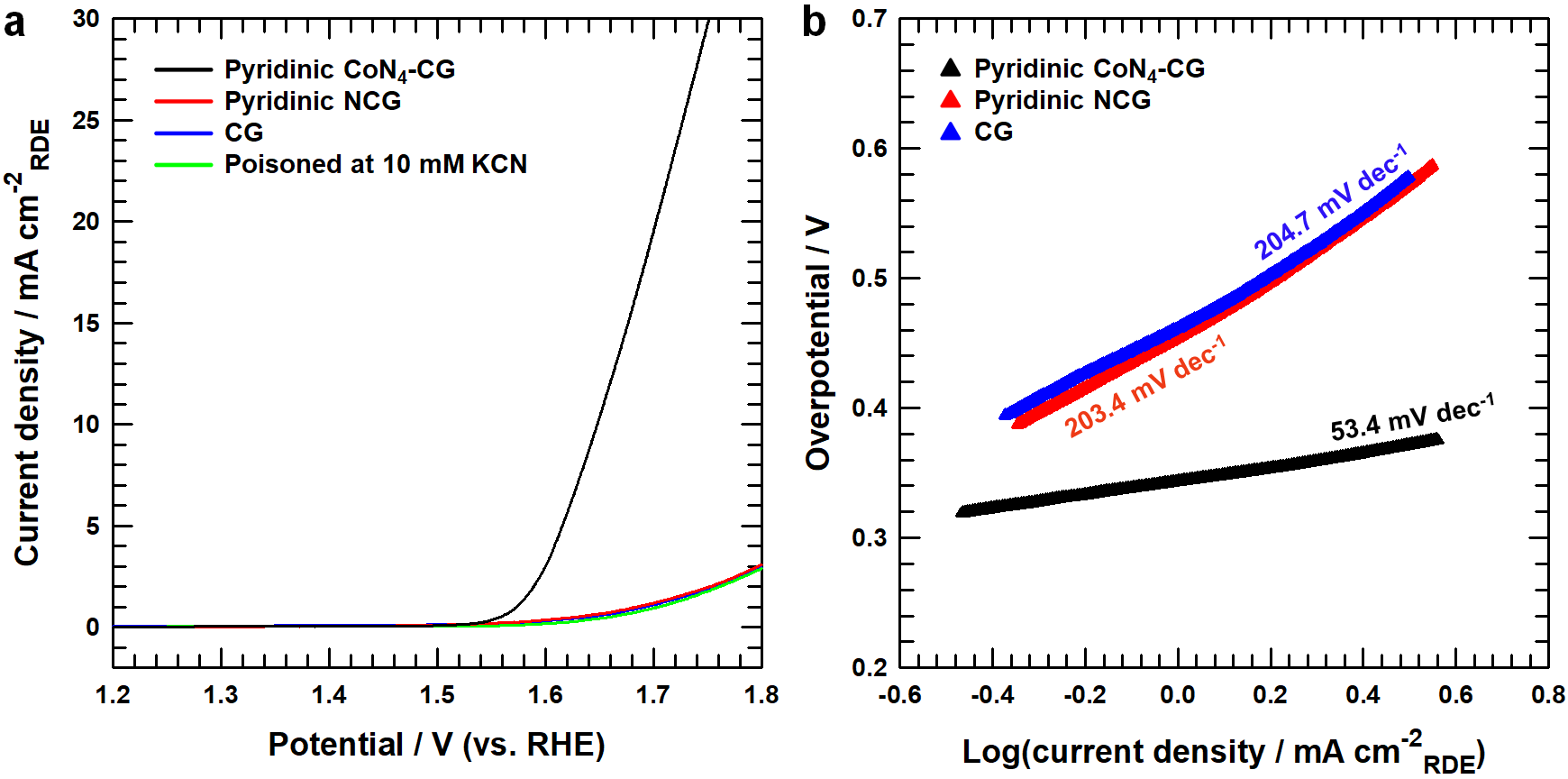


**Figure S13.** (a) *iR*-corrected OER polarization curves of Pyridinic CoN_4_-CG, Pyridinic NCG, CG, and KCN-poisoned Pyridinic CoN_4_-CG at a scan rate of 5mV s^-1^ and a rotation rate of 1600 rpm in O_2_-saturated 0.5M H_2_SO_4_ electrolyte. (b) Tafel plots for Pyridinic CoN_4_-CG, Pyridinic NCG, and CG recorded at a scan rate of 5mV s^-1^ and a rotation rate of 1600 rpm in O_2_-saturated 0.5M H_2_SO_4_ electrolyte.


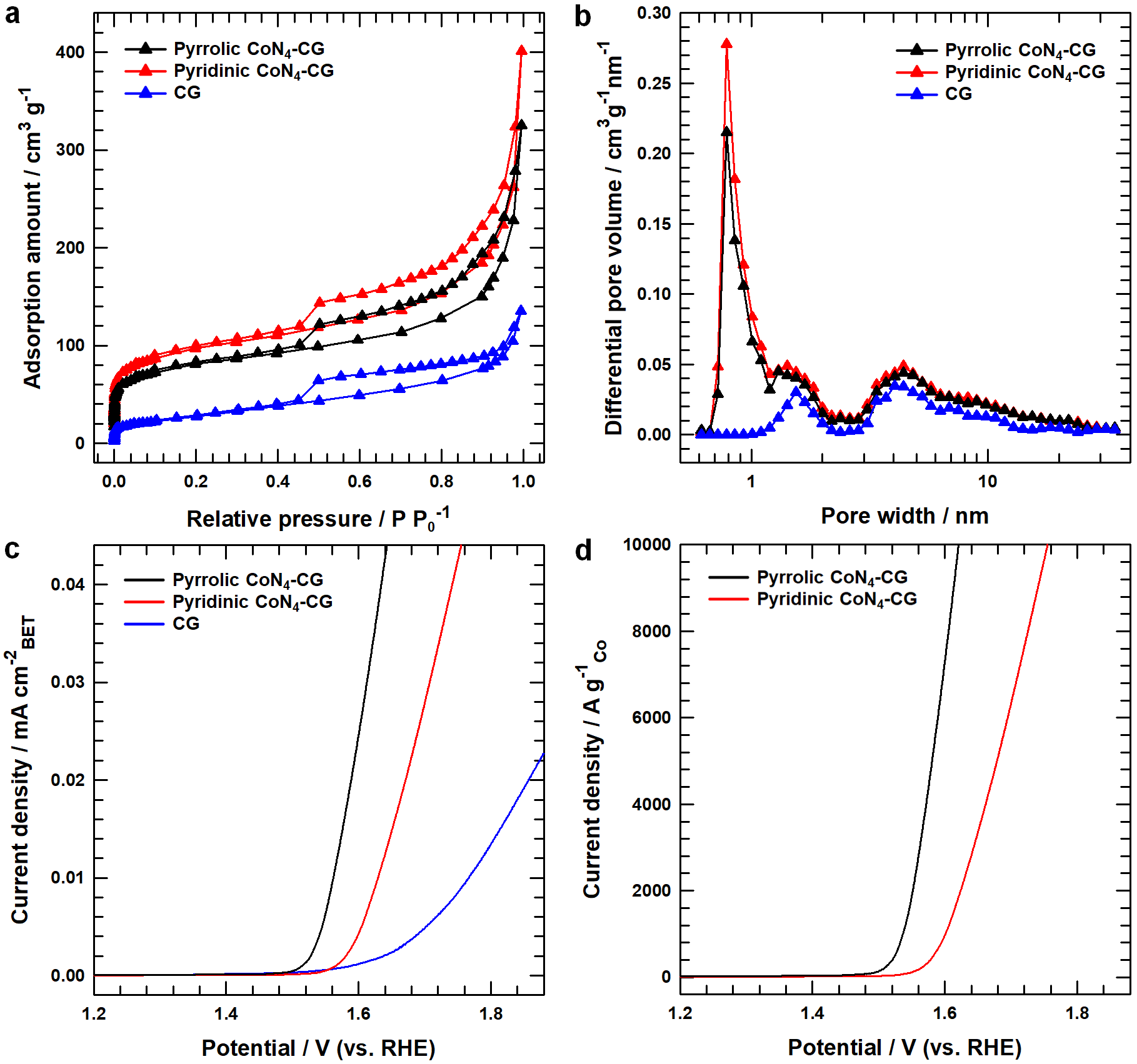


**Figure S14.** (a) N_2_ adsorption-desorption isotherms of Pyrrolic CoN_4_-CG, Pyridinic CoN_4_-CG, and CG. (b) Pore size distribution of Pyrrolic CoN_4_-CG, Pyridinic CoN_4_-CG, and CG. The micro and mesoporous features were confirmed via N_2_ adsorption-desorption measurements. Brunauer-Emmett-Teller (BET) and nonlocal density functional theory (NLDFT) analysis were employed to investigate the pore structure of Pyrrolic CoN_4_-CG, Pyridinic CoN_4_-CG, and CG. All the samples show the sharp increase region at low P/P_o_ below 0.2 and hysteresis loop after P/P_o_ = 0.4 in N_2_ adsorption-desorption isotherm, demonstrating the coexistence of micro and mesopores (Figure S14a). The BET specific surface areas for Pyrrolic CoN_4_-CG, Pyridinic CoN_4_-CG, and CG were determined to be 276.3, 331.5, and 102.4 m^2^ g^-1^, respectively, and the pore volumes for Pyrrolic CoN_4_-CG, Pyridinic CoN_4_-CG, and CG were calculated to be 0.514, 0.558, and 0.269 cm^3^ g^-1^, respectively. Importantly, Pyridinic CoN_4_-CG shows the dramatic increase of sharp increase region at low P/P_o_ below 0.2 in N_2_ adsorption-desorption isotherm and integrated micropore region in pore size distribution compared to CG. The introduction of Co single atomic site on CG occur the elimination of sp^3^ carbon atoms in graphene carbon network, forming the defect sites on graphene layers. Then the integration of these neighboring defect sites causes the formation of micropores, resulting in the significant increase of total specific surface area and pore volume. Moreover, Pyridinic CoN_4_-CG exhibits the increased micropore surface area and pore volume due to the formation of Co single atomic center with pyridinc N ligand. These pyridinc N ligands which can provide stable covalent bond between C and Co enable the thermodynamically stable pyridinic CoN_4_ moiety, resulting in the favorable environment for the introduction of Co single atomic center. As a result, the large amount of introduced single atomic Co can create the larger population of defect sites on CG, and the integration of these defect sites leads to the increase of micropore surface area and pore volume. However, Pyrrolic CoN_4_-CG shows the slightly decreased micropore surface area and pore volume than those of Pyridinic CoN_4_-CG since pyrrolic N ligand is thermodynamically unstable than pyridinic N ligand. Therefore, the pyrrolic N causes the unfavorable environment in which the pyrollic CoN_4_ moieties are difficult to be formed, and these trends show a good agreement in inductively coupled plasma atomic emission spectroscopy (ICP-AES) analysis. The weight percentage of Co in Pyrrolic CoN_4_-CG, and Pyridinic CoN_4_-CG measured by ICP-AES is 0.93 and 1.46 wt%, respectively. (c) LSV polarization curves normalized by BET specific surface area for Pyrrolic CoN_4_-CG, Pyridinic CoN_4_-CG, and CG. (d) LSV polarization curves normalized by Co loading mass for Pyrrolic CoN_4_-CG and Pyridinic CoN_4_-CG. Considering the catalyst loading mass on RDE and Co weight percentage, the loading mass of single atomic Co on RDE for Pyrrolic CoN_4_-CG and Pyridinic CoN_4_-CG is 1.98 and 3.08 μg_co_ cm^-2^_RDE_, respectively.


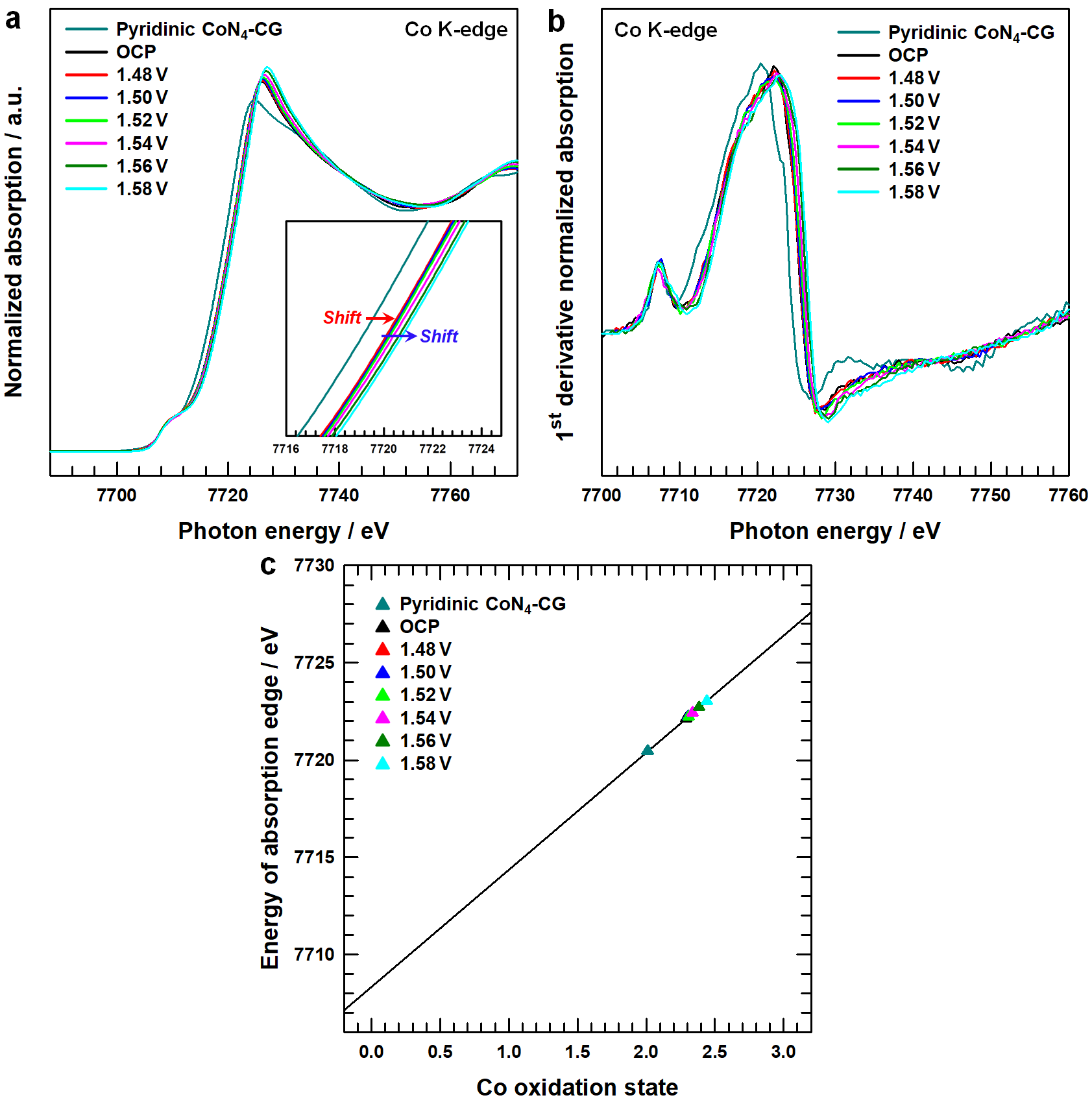


**Figure S15.** (a) Operando Co K-edge XANES spectra of Pyridinic CoN_4_-CG from 1.48 to 1.58 V vs. RHE in O_2_-saturated 0.5M H_2_SO_4_ electrolyte. (b) Corresponding first derivative of Co K-edge XANES absorption edge. (c) Plot of absorption edge position vs Co valence state for Pyridinic CoN_4_-CG at different applied potentials.


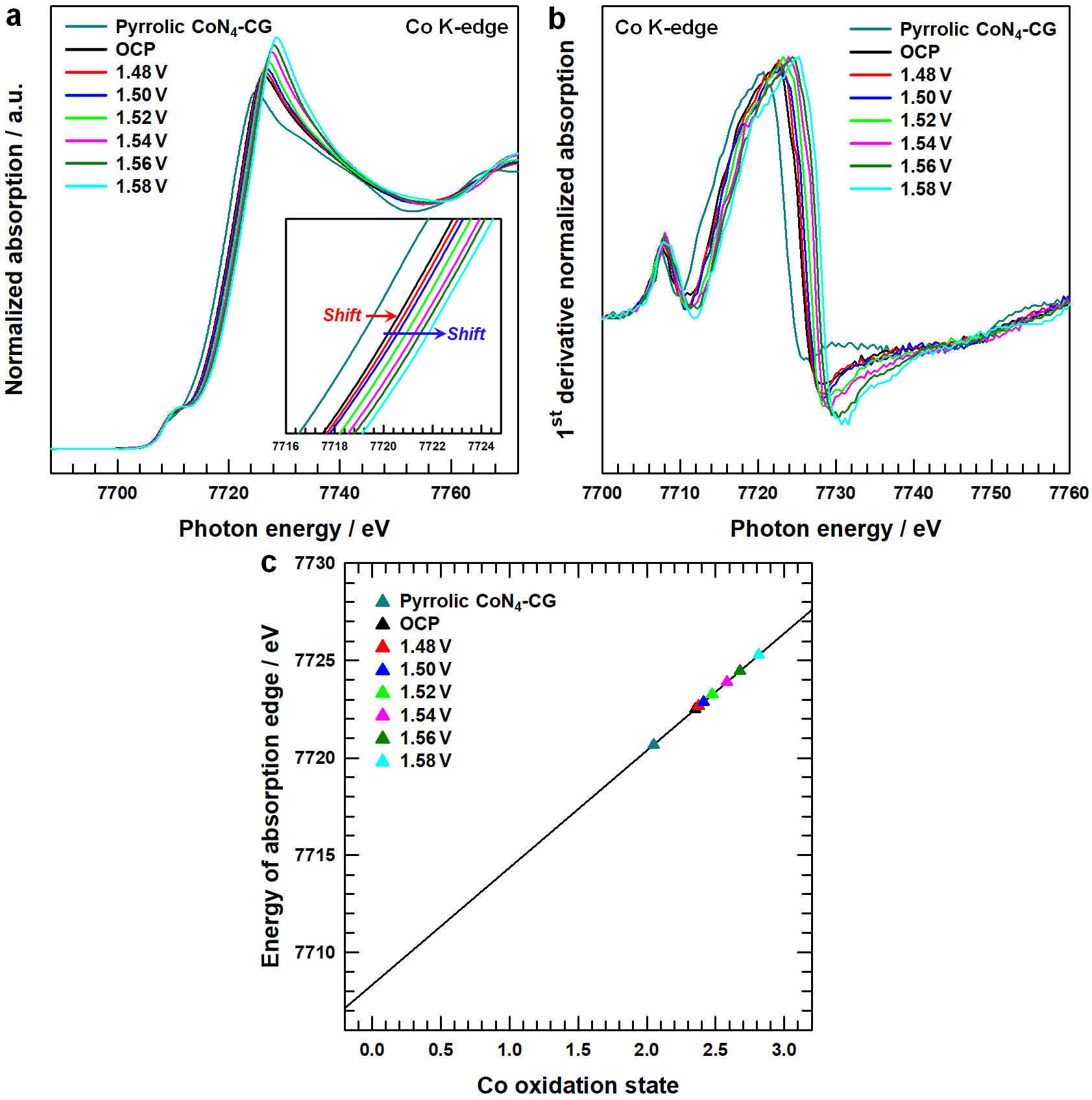


**Figure S16.** (a) Operando Co K-edge XANES spectra of Pyrrolic CoN_4_-CG from 1.48 to 1.58 V vs. RHE in O_2_-saturated 0.5M H_2_SO_4_ electrolyte. (b) Corresponding first derivative of Co K-edge XANES absorption edge. (c) Plot of absorption edge position vs Co valence state for Pyrrolic CoN_4_-CG at different applied potentials.


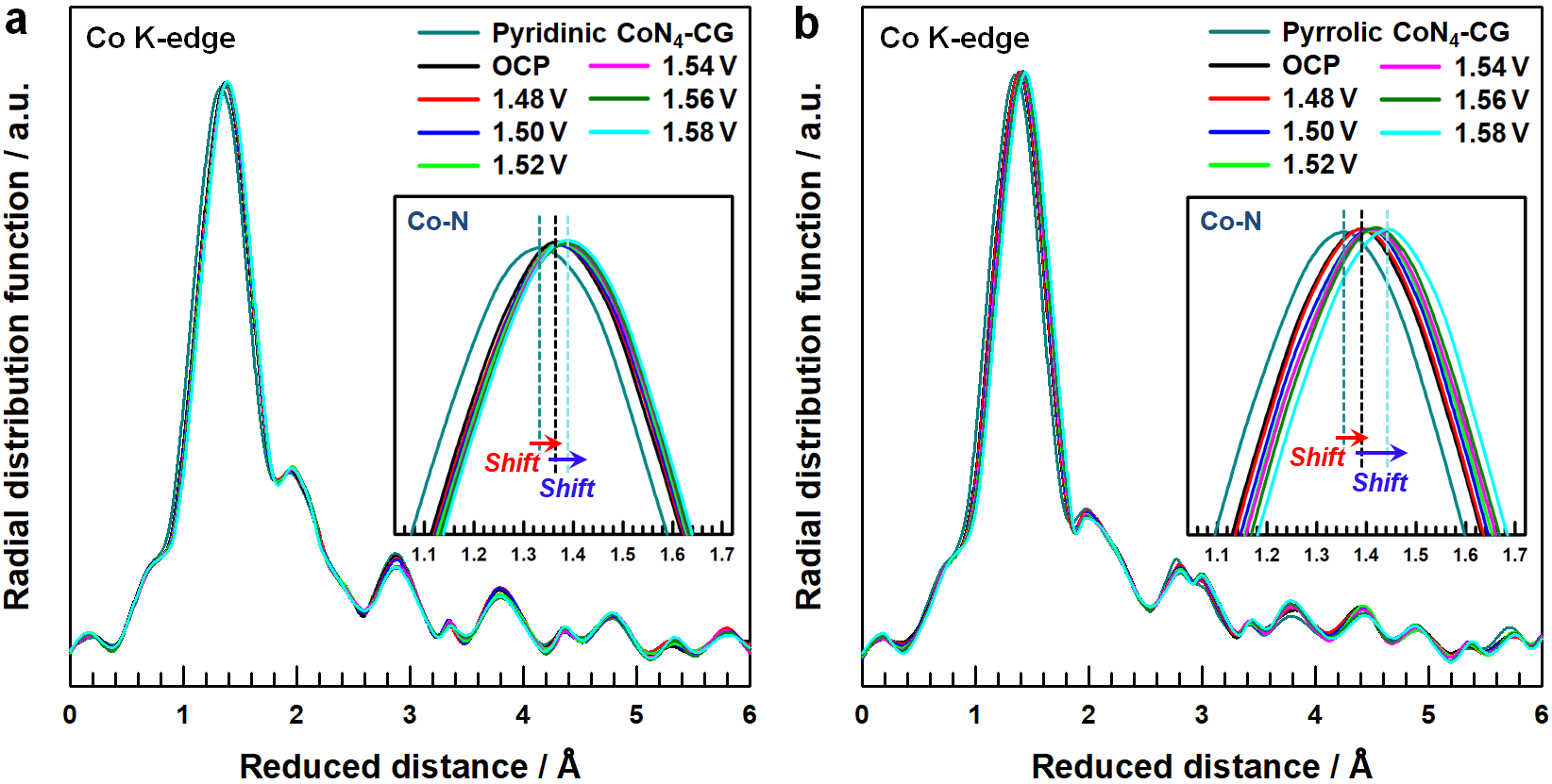


**Figure S17.** Operando Co K-edge EXAFS spectra from 1.48 to 1.58 V vs. RHE in O_2_-saturated 0.5M H_2_SO_4_ electrolyte for (a) Pyridinic CoN_4_-CG and (b) Pyrrolic CoN_4_-CG.


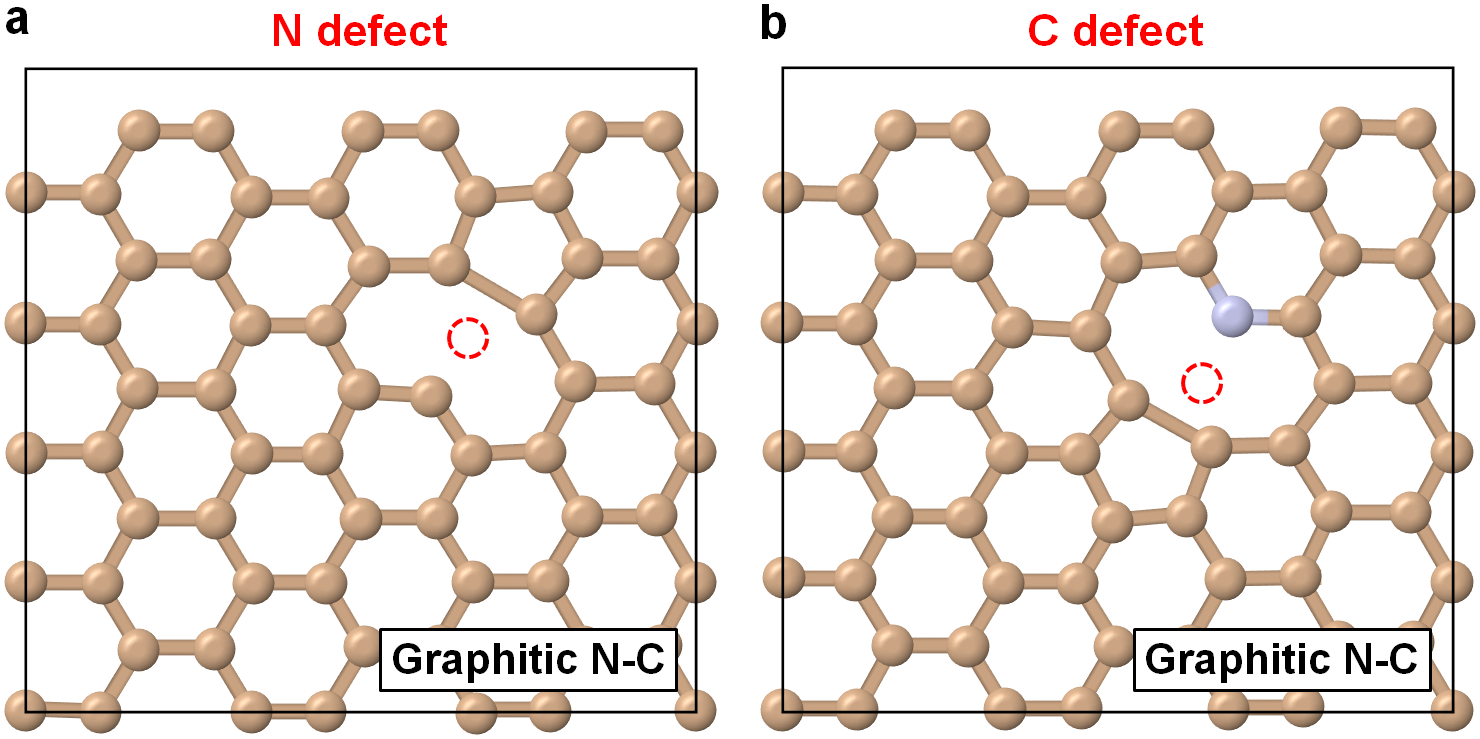


**Figure S18.** Atomic defect model system of (a) N defect and (b) C defect in graphitic N doped carbon network.

**Table S1.** Atomic ratio percentage (at.%) of the specific nitrogen species in the samples calculated from XPS N 1s spectra.

| **Sample** | **Pyridinic N (at.%)** | **Pyridinic N-Co (at.%)** | **Pyrrolic N (at.%)** | **Pyrrolic N-Co (at.%)** | **Graphitic N (at.%)** |
| --- | --- | --- | --- | --- | --- |
| **Pyrrolic CoN_4_-CG** | 41.5 | 0 | 18.3 | 9.8 | 30.4 |
| **Pyrrolic CoN_4_-CG**  **_after CP** | 44.0 | 0 | 9.5 | 10.9 | 35.6 |
| **Pyridinic CoN_4_-CG** | 47.0 | 14.7 | 3.0 | 0 | 35.4 |
| **Pyridinic CoN_4_-CG _after CP** | 49.0 | 10.9 | 1.7 | 0 | 38.5 |

The atomic ratios (at.%) of nitrogen species were quantified based on the deconvoluted peak area ratios obtained from XPS N 1s spectra shown in Figure 3f for pristine Pyrrolic CoN_4_-CG and Pyridinic CoN_4_-CG samples, and in Figure 5f for their post-chronopotentiometry counterparts (Pyrrolic CoN_4_-CG_after CP and Pyridinic CoN_4_-CG_after CP).

**Table S2.** Weight raito percentage (wt.%) of cobalt and specific nitrogen species in the samples.

| **Sample** | **Co (wt%)** | **Total N (wt%)** | **Pyridinic N (wt%)** | **Pyridinic N-Co (wt%)** | **Pyrrolic N (wt%)** | **Pyrrolic N-Co (wt%)** | **Graphitic N (wt%)** |
| --- | --- | --- | --- | --- | --- | --- | --- |
| **Pyrrolic CoN_4_-CG** | 0.93 | 9.22 | 3.83 | 0 | 1.68 | 0.90 | 2.81 |
| **Pyrrolic CoN_4_-CG_after CP** | 0.99 | 8.81 | 3.88 | 0 | 0.83 | 0.96 | 3.14 |
| **Pyridinic CoN_4_-CG** | 1.46 | 9.60 | 4.51 | 1.41 | 0.29 | 0 | 3.39 |
| **Pyridinic CoN_4_-CG_after CP** | 1.08 | 9.56 | 4.68 | 1.04 | 0.16 | 0 | 3.68 |

The Co content (wt.%) in the samples was determined using inductively coupled plasma atomic emission spectroscopy (ICP-AES), while the nitrogen species distribution (wt.%) was calculated by combining elemental analysis (EA) and XPS N 1s spectral data. The weight percentages of individual nitrogen species were obtained by multiplying their respective atomic percentages (at.%, Table S1) by the total nitrogen content (wt.%) from EA measurements.

**Table S3.** Actual nitrogen species loading mass (μg/cm^2^) calculated from catalyst loading mass and nitrogen composition (wt%) from Table S2.

| **Sample** | **Total N (μg/cm^2^)** | **Pyridinic N (μg/cm^2^)** | **Pyridinic N-Co (μg/cm^2^)** | **Pyrrolic N (μg/cm^2^)** | **Pyrrolic N-Co (μg/cm^2^)** | **Graphitic N (μg/cm^2^)** |
| --- | --- | --- | --- | --- | --- | --- |
| **Pyrrolic CoN_4_-CG** | 19.632 | 8.152 | 0 | 3.583 | 1.921 | 5.976 |
| **Pyrrolic CoN_4_-CG_after CP** | 16.604 | 7.31 | 0 | 1.57 | 1.813 | 5.911 |
| **Pyridinic CoN_4_-CG** | 20.246 | 9.516 | 2.966 | 0.606 | 0 | 7.158 |
| **Pyridinic CoN_4_-CG_after CP** | 18.318 | 8.967 | 1.992 | 0.305 | 0 | 7.054 |

The catalyst loading on the glassy carbon electrode was 0.213 and 0.211 mg_catalyst_ cm^-2^ for Pyrrolic CoN_4_-CG and Pyridinic CoN_4_-CG, respectively. Post-OER stability testing, the loading masses for Pyrrolic CoN_4_-CG_after CP and Pyridinic CoN_4_-CG_after CP were determined to be 0.189 and 0.191 mg_catalyst_ cm^-2^, respectively, by measuring the weight difference between catalyst-loaded and pristine electrodes. The actual loading masses of total nitrogen and individual nitrogen species were calculated by multiplying the catalyst loading mass by the weight percentage of each nitrogen species (Table S2). Based on these values, the mass loss rates of each nitrogen species for Pyrrolic CoN_4_-CG and Pyridinic CoN_4_-CG after OER were calculated and presented in Figure 5g.

**References**

1. G. Kresse, J. Hafner, *Phys. Rev. B* **1993**, *47* (1), 558.
2. *G. Kresse, J. Hafner, Phys. Rev. B* **1994**, *49* (20), 14251.
3. G. Kresse, J. Furthmüller, *Phys. Rev. B* **1996**, *54* (16), 11169.
4. G. Kresse, J. Furthmüller, *Comput. Mater. Sci.* **1996**, *6* (1), 15.
5. J. P. Perdew, K. Burke, M. Ernzerhof, *Phys. Rev. Lett.* **1996**, *77* (18), 3865.
6. G. Kresse, D. Joubert, *Phys. Rev. B* **1999**, *59* (3), 1758.
7. J. Heyd, G. E. Scuseria, M. Ernzerhof, *J. Chem. Phys.* **2003**, *118* (18), 8207.
8. S. Grimme, J. Antony, S. Ehrlich, H. Krieg, *J. Chem. Phys.* **2010**, *132* (15).
9. J. Börgel, M. G. Campbell, T. Ritter, *J. Chem. Educ.* **2016**, *93* (1), 118.
